# Bavachin Protects Human Aortic Smooth Muscle Cells Against *β*-Glycerophosphate-Mediated Vascular Calcification and Apoptosis *via* Activation of mTOR-Dependent Autophagy and Suppression of *β*-Catenin Signaling

**DOI:** 10.3389/fphar.2019.01427

**Published:** 2019-12-19

**Authors:** Hu-Qiang He, Betty Yuen Kwan Law, Ni Zhang, Cong-ling Qiu, Yuan-Qing Qu, An-Guo Wu, Yu Han, Qi Song, Wen-Lu Zheng, Yong Liu, Yan-Zheng He, Vincent Kam Wai Wong

**Affiliations:** ^1^Faculty of Chinese Medicine, Macau University of Science and Technology, Macau, China; ^2^State Key Laboratory of Quality Research in Chinese Medicine, Macau University of Science and Technology, Macau, China; ^3^Department of Vascular Surgery, Affiliated Hospital of Southwest Medical University, Luzhou, China; ^4^Laboratory of Chinese Materia Medical, Department of Pharmacology, School of Pharmacy, Southwest Medical University, Luzhou, China; ^5^Institute of Cardiovascular Research, The Key Laboratory of Medical Electrophysiology, Ministry of Education of China, Collaborative Innovation Center for Prevention and Treatment of Cardiovascular Disease of Sichuan Province, Southwest Medical University, Luzhou, China; ^6^Department of Thoracic and Cardial Surgery, Affiliated Hospital of Southwest Medical University, Luzhou, China; ^7^Department of Nuclear Medicine, Affiliated Hospital of Southwest Medical University, Luzhou, China; ^8^Nuclear Medicine and Molecular Imaging Key Laboratory of Sichuan Province, Affiliated Hospital of Southwest Medical University, Luzhou, China

**Keywords:** vascular calcification, autophagy, Wnt/β-catenin, Apoptosis, Atg7

## Abstract

Vascular calcification is a major complication of cardiovascular disease and chronic renal failure. Autophagy help to maintain a stable internal and external environment that is important for modulating arteriosclerosis, but its pathogenic mechanism is far from clear. Here, we aimed to identify the bioactive compounds from traditional Chinese medicines (TCM) that exhibit an anti-arteriosclerosis effect. In β-glycerophosphate (β-GP)-stimulated human aortic smooth muscle cells (HASMCs), the calcium level was increased and the expression of the calcification-related proteins OPG, OPN, Runx2, and BMP2 were all up-regulated, followed by autophagy induction and apoptosis. Meanwhile, we further revealed that β-GP induced apoptosis of human osteoblasts and promoted differentiation of osteoblasts through Wnt/β-catenin signaling. Bavachin, a natural compound from *Psoralea corylifolia*, dose-dependently reduced the level of intracellular calcium and the expression of calcification-related proteins OPG, OPN, Runx2 and BMP2, thus inhibiting cell apoptosis. In addition, bavachin increased LC3-II and beclin1 expression, along with intracellular LC3-II puncta formation, which autophagy induction is Atg7-dependent and is regulated by suppression of mTOR signaling. Furthermore, addition of autophagy inhibitor, wortmannin (WM) attenuated the inhibitory effect of bavachin on β-GP-induced calcification and apoptosis in HASMCs. Collectively, the present study revealed that bavachin protects HASMCs against apoptosis and calcification by activation of the Atg7/mTOR-autophagy pathway and suppression of the β-catenin signaling, our findings provide a potential clinical application for bavachin in the therapy of cardiovascular disease.

## Introduction

Vascular calcification (VC) is the pathogenic process of accumulating the crystals of calcium phosphate with in the blood vessel (Hofmann Bowman and McNally, 2012; Sun et al., 2012; Yahagi et al., 2017; Chellan et al., 2018; Benjamin et al., 2019).VC has been recognized as a marker of atherosclerotic plaques, which involves a complex pathological process, and has very high morbidity and mortality (Thompson and Towler, 2012; Asmat et al., 2016; Kay et al., 2016). Vascular smooth muscle cells (VSMCs) play a pivotal role in arteriosclerosis. Previous studies have reported that VC is a passive process resulting from the accumulation of calcium phosphate (Thompson and Towler, 2012). However, VC is currently considered as an active cell-based transition process converting VSMC phenotype to osteoblasts (Li et al., 2019). However, VC is resulted from a complex pathophysiological process, comprising several different mechanisms, including hypercalcemia, inflammatory cytokines, oxidative stress, etc. Cai et al. (2016) reported that Wnt/β-catenin signaling enhances osteogenic differentiation and calcification of VSMCs by direct up-regulation of Runx2.

Increasing studies correlated the relationship between cellular apoptosis and calcification of VSMCs. For instance, apoptotic cell death was found in both human and animal atherosclerotic plaques (Kapustin et al., 2011), suggesting that apoptosis promotes calcification of the matrix, mainly through the release of apoptotic bodies, combined with nucleation sites of VC. In uremia calcification model, there is a positive correlation between apoptosis and calcification of VSMCs. In addition, apoptosis always occurs before calcification, in which apoptotic bodies were found to contain high concentrations of calcium, to deposit on extracellular matrix (ECM), and eventually leading to calcification (Rodriguez et al., 2014). For example, the release of calcified vesicles and the deposition of calcification can be significantly reduced by inhibiting cell apoptosis through Caspase inhibitors. In the model of Hyperphosphate-induced VC, inhibition of calcium aggradation can be achieved by preventing apoptosis and potentiating autophagy (Ciceri et al., 2016).

Induction of autophagy has been commonly found in several cardiovascular diseases (CD), however, whether the activation of autophagy would play a protective or harmful effect on the vascular system is required to be elucidated (Choi et al., 2013). Accumulating evidence revealed that apoptosis and autophagy could simultaneously induce and influence the cell fates (Abdel-Mohsen et al., 2018). Autophagy is a “recycling” cellular process by lysosome-mediated degradation and turnover of damaged cytosolic material, which process is tightly regulated by several highly conserved autophagy-related (ATG) genes (Grootaert et al., 2018). Autophagy plays a crucial role in phenotype transition and oxidative stress of VSMC cells (Mialet-Perez and Vindis, 2017). Frauscher reported that autophagy is an endogenous response of VSMC to prevent calcification in uremia (Frauscher et al., 2018). Rapamycin-mediated autophagy was found to protect the cells and mice suffered from uremia-mediated calcification, probably by inhibition of osteogenic trans-differentiation of VSMCs (Frauscher et al., 2018). Indeed, induction of autophagy would modulate the functions of the vessel wall and participate in the initiation and progression of vascular diseases such as arteriosclerosis (AS). As a result, autophagy is not only considered as a self-protective mechanism, but also induces autophagic cell death in VC (Wei et al., 2014).

Bavachin is a phytoestrogen isolated from herb called *Psoralea corylifolia*. Bavachin has been indicated to have anti-cancer, anti-inflammatory, anti-bacterial, lipid-lowering and cholesterol-reducing effects (Takeda et al., 2018). Song reported that bavachalcone from *Cullen corylifolium* induces autophagy in HepG2 cells (Song et al., 2018). We found that bavachin can activate autophagy in human aortic smooth muscle cells (HASMCs). Nevertheless, autophagy may have a protective effect by attenuating the calcification of VSMCs (Dai et al., 2013).

In this study, we used β-GP to induce a calcification process in HASMCs. We determined the effect of bavachin on β-GP-induced calcification and apoptosis in VSMCs and explored the mechanistic pathways involved. This study showed, for the first time, that bavachin can protect HASMCs against apoptosis and calcification by induction of the Atg7/mTOR dependent autophagy pathway and inhibition of the Wnt/β-catenin pathway.

## Materials and Methods

### Cells Culture

Primary HASMCs (human aortic smooth muscle cells) were obtained from ScienCell Research-Laboratories (USA). DMEM medium (Gibco, Waltham, MA, USA) was supplied with 10% FBS, and 1% PSG (Gibco, Waltham, MA, USA) was used as the culture medium. The cell was cultured in an incubator at 37 °C with 5% humidified CO_2_.

### Experimental Reagents and Instruments

The concentration of each reagent and antibodies listed below is described in the result section. Bavachin was purchased from PUSH BIO (Cheng Du, China). siRNA against human Atg7 was synthesized by GeneChem (Shanghai, China).

### Primary Antibodies

LC3-II, Beclin-1, p62, p-mTOR, β-catenin, Caspase9, Caspase3, Bax, Bak, and Bcl-2 were obtained from Cell Signaling Technologies (Danvers, USA). OPN, BMP2, Runx2 were purchased from ABGENT (Nanjing, China). OPG, α-SMA and β-actin were acquired from GeneTex (Texas, USA), Biolegend (Peking, China), and Santa Cruz (MO, USA), respectively.

### Secondary Antibodies

ZyMaxTM TRITC-conjugated anti-mouse and ZyMaxTM FITC-conjugated anti-rabbit antibodies were purchased from Invitrogen (Invitrogen, USA), and HRP-conjugated antibody was acquired from Cell Signaling Technologies (Danvers, USA).

### Staining Reagents

VON KOSSA Staining Kit (Genmed, Shanghai, China). 3-(4,5-dimethylthiazol-2-yl)-2,5-diphenyl-tetrazolium bromide (MTT), Fluo-3 (Sigma, USA) dye, rhodamine-phalloidin (Sigma, MO, USA), wortmannin (WM), and β-glycerophosphate were purchased from Sigma (St. Louis, USA) respectively.

### Cytotoxicity Assay

Cells viability was determined by the half-maximal inhibitory concentration (IC_50_) using MTT (0.5mg/ml) assay, as previously described (Ulukaya et al., 2008). Briefly, 4x10^3^ cells were cultured/well in 96-well plate and exposed to bavachin dissolved in dimethyl sulfoxide (DMSO) at a different concentration (from 0 to 100 µM) for 72 h, whereas cells receiving no treatment were served as control. The samples were then incubated with MTT for 4 h at 37°C followed by overnight incubation of special solubilization buffer (10%SDS in 0.01Mol/L HCL). A_570_ nm was then determined in each well by a microplate reader (Tecan Infinite M200 PRO, Tecan, Männedorf, Switzerland). Cell viability was calculated as following: Percentage of Cells viability = (*A*_treated_ − *A*_background_)/(*A*_control_ − *A*_background_) × 100.

### VON KOSSA Staining for Calcification Detection

HASMCs in the logarithmic phase were cultured on the cover slip in 6-well plate and divided into four treatment groups (n = 3 for each group): untreated control, β-GP treatment, and co-treatment of β-GP with different concentration of Bavachin (12.5 µM and 25 µM). β-GP and Bavachin were directly dissolved in the culture medium. Each experimental group was cultured at 37°C for 14 consecutive days. VON KOSSA staining Kit was then used for the detection of the deposition level of calcium in the cellular models according to the manual from the manufacturer. In brief, the cells were washed with CENMED cleaning solution and fixed with 4% paraformaldehyde for 10 min. Two hundred microliters of CENMED staining solution (containing 1% silver nitrate solution) was then added to the cells followed by exposure to sunlight at room temperature until black deposits of calcium were observed. Finally, the cells were further incubated with CENMED cleaning solution for 2 min after the staining solution was removed and immediately observed under an optical microscope for image collection (Olympus, Japan).

### Flow Cytometry Analysis for Intracellular Calcium Level and Apoptosis

Cells belonging to the untreated control, β-GP-treated group, and β-GP/bavachin (12.5 µM or 25 µM)-treated group were cultured for 72 h in 6-well plate, then detached with 0.25% Trypsin-EDTA. For the detection of cytotoxicity, cells were stained with propidium iodide (50 µg/ml) and annexin V-FITC (2.5 µg/ml) at RT for 15 min with FITC Annexin V Apoptosis Detection Kit (BD, USA) in the dark as described previously (Saadat et al., 2015). While the cytosolic calcium level of the cells was detected by the addition of Fluo-3 dye at RT for 30 min. Ultimately, samples diluted with 300 µl binding-buffer or sheath fluid were detected by flow cytometer (BD FACSAria III, USA). Data were acquired, analyzed, and performed with CellQuest (BD Biosciences, USA)

### Immunofluorescence Detection

Cells (2 × 10^5^ cells/well) seeded on the cover slip in 6-well plate were incubated overnight and treated with the untreated control, β-GP treatment, and co-treatment of β-GP and Bavachin (25 µM) with or without WM for 72 h. After 20 min fixation with 4% paraformaldehyde, the cells were permeabilized with methanol at RT for 5 min. The fixed cells were co-stained with mouse anti-LC3 (1:200) and rabbit anti-OPN (1:200) or rhodamine-phalloidin (1:200) in blocking buffer (5% BSA-TBST) overnight at 4°C. After washing, slides were incubated with TRITC anti-mouse antibody (1:200) and FITC anti-rabbit antibody (1:200) at 37°C for 1 h. After washing with ice-cold PBS, the cells were then stained with DAPI (Beyotime, USA) for 5 min. Finally, the cover slips were mounted onto microslides by FluorSave^™^ medium (Calbiochem, USA) for images capturing with Photometrics CoolSNAP HQ2 CCD camera on the Olympus IX71-Applied Precision Delta-Vision restoration microscope (Applied Precision, USA).

### Western Blotting

Cells were lysed with RIPA lysate (Cells Signaling Technology, USA) for cellular proteins extraction with the concentrations determined by Bio-Rad protein assay kit (Hercules, USA). Proteins in SDS/PAGE were transported to the chemiluminescent film (GE Healthcare, Buckinghamshire, UK) by electrophoresis. Then the primary and secondary antibody at a concentration of (1:1,000) and (1:2,500) were used, respectively. Protein was detected by Clarity Western ECL (Bio-Rad, USA) and Amersham Imager 600 (GE Healthcare, Buckinghamshire, UK). The software image J (NIH, USA) was used for quantifying the band intensity. The expression level of targeted proteins was quantified by normalization with the expression of β-actin and represented as fold change relative to the control group.

### Quantification of Calcium Deposition and Intracellular Calcium Measurement

The calcification of HASMCs was stimulated by β-GP for three days in 6-well plate, and the formation of calcified black spots was quantified by inverted microscopy under 4× magnification. The cellular calcium content was further determined as reported previously (Torremadé et al., 2016). Briefly, Cell lysate was collected from HASMCs and decalcified by HCL. The amount of the protein content was evaluated by using a protein assay kit (Bio-Rad, USA). While the calcium content was quantified by the O-cresolphthalein complexone assay (Changcheng, China). The relative amount of cytosolic calcium in the sample was then calculated by the formula: *calcium content (µg)/protein content (mg)*. The cytosolic calcium was also determined by using the FLIPR Calcium 6 Assay Kit (Molecular Devices, USA) to examine the cells treated with β-GP or β-GP/bavachin (12.5 µM or 25 µM) by observing of calcium deposits under the fluorescence microscope.

### Statistical Approach

All the data were statistically processed using SPSS 20.0 software package. Data were analyzed with Student’s two-tailed t-test or one-way ANOVA. The analysis of variance homogeneity was performed first. If the variances were equal, the Fisher’s Least Significant Difference (LSD) test was performed. Otherwise, the Games–Howell test was performed. Measurement of data such as apoptotic ratio was expressed as mean ± S.D., n = 3. Graphs were plotted using GraphPad Prism 5.0 software. *P* < 0.05 was considered as statistically significant.

## Results

### β-GP Induces Vascular Calcification in HASMCS

**Figure 1:**
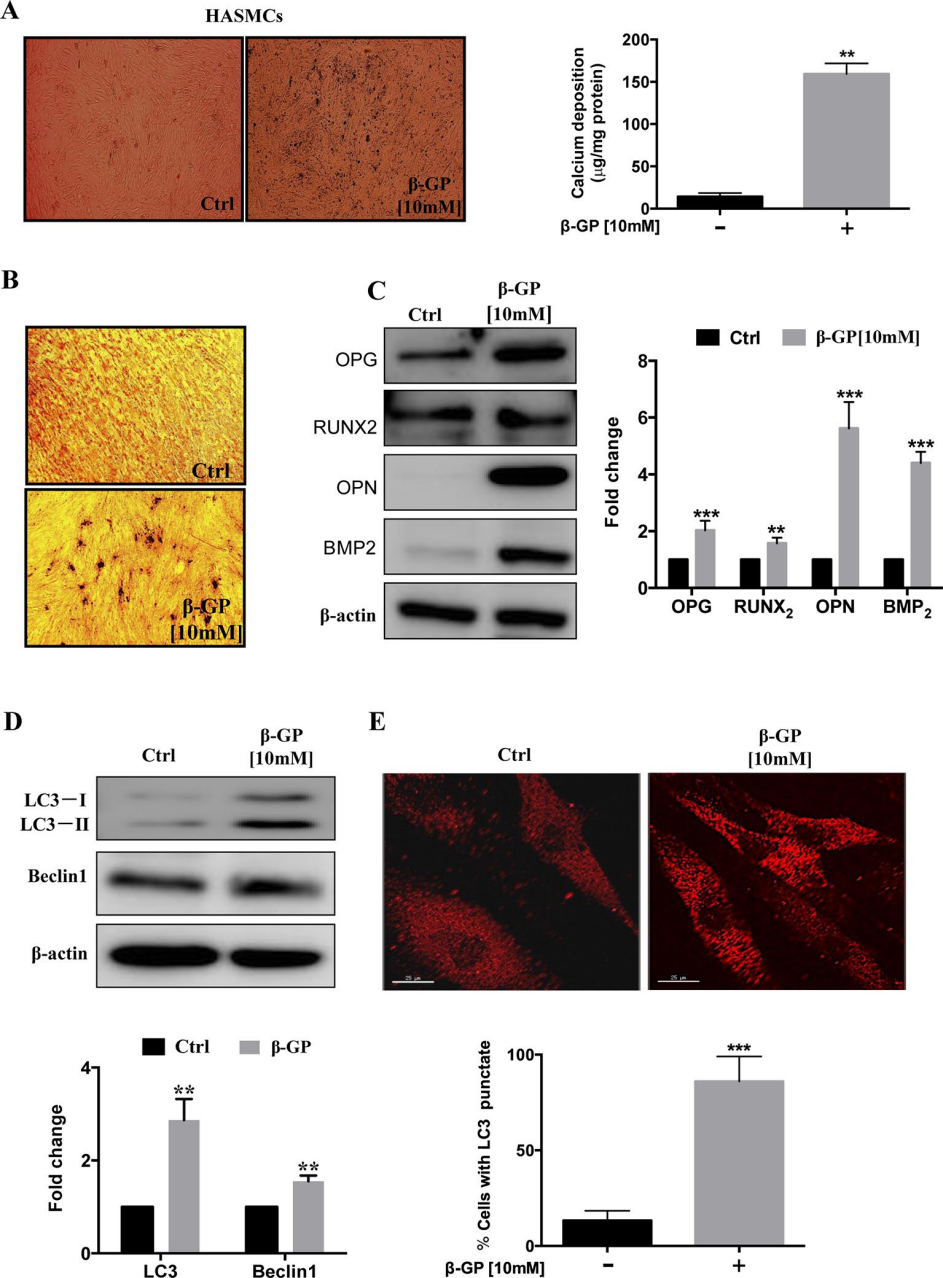
β-GP induces calcification in HASMCs. **(A)** HASMCs were treated with DMSO or 10 mM β-GP for 3 days. Cell calcified black spots levels were observed using general microscopy (black spots), magnification 4×; Quantification of calcium levels using the O-cresolphthalein complexone method. **(B)** Ca^2+^ deposition was visualized at the light microscopy after VON KOSSA staining. No calcium deposits were found in the control culture, while β-GP promoted calcium deposition; magnification10×. Representative images were shown from three independent experiments. **(C)** Expression of calcification-related proteins (OPG, Runx2, OPN, and BMP2) were analyzed by Western blotting. The full-length images of Western blot are shown in **Figure S1C**. **(D)** Expression of autophagy-related proteins (LC3-I/II and Beclin1) were analyzed by Western blotting. The full-length images of Western blot are shown in **Figure S1D**. **(E)** Autophagosomes were indicated by the fluorescent puncta (red); scale bar = 25 µm. All the HASMC lysates in the Western blot assay were collected at 72 h after treatment with10 mM β-GP. Statistical significance was analyzed by the t-test (**p < 0.01, ***p < 0.001). The data is represented as mean ± S.D. (n = 3).

To establish the vascular calcification model, we stimulated HASMCs with β-GP for 3 days, and the calcium deposition (black spots) were induced in HASMCs (Figure 1A, *p <0.001*). Also, HASMCs were stimulated with β-GP for 14 days; and more black-stained deposits were observed after VON KOSSA staining (Figure 1B). Since VC is an organized and highly-regulated process in comparison with bone mineralization. And numerous studies demonstrated that mineralized matrix and Runx2 are the key phenotypic markers for osteoblasts, in which expression are usually up-regulated during osteoblastic differentiation in HASMCs (Liu et al., 2016). Here, our results demonstrated that the expression levels of calcification-related proteins OPG, OPN, Runx2, and BMP2 were elevated in comparison to the control group (Figure 1C, *p < 0.01*), which coincided with other findings that β-GP induces calcification and osteoblast-like differentiation of VSMCs (Peng et al., 2017). To investigate whether β-GP can induce autophagy in HASMCs, we treated HASMCs with β-GP and observed whether β-GP could activate autophagy by immunoblotting and immunofluorescence. Firstly, the expression of Beclin1 and LC3-I/II were detected by Western blotting and results indicated that these two autophagic markers were up-regulated in HASMCs after β-GP induction (Figure 1D, *p < 0.01*). Meanwhile, the TRITC–LC3 fluorescence puncta were markedly increased from 3 days in β-GP-treated HASMCs (Figure 1E, *p < 0.001*).

### Bavachin Induces Autophagy in HASMCs

**Figure 2:**
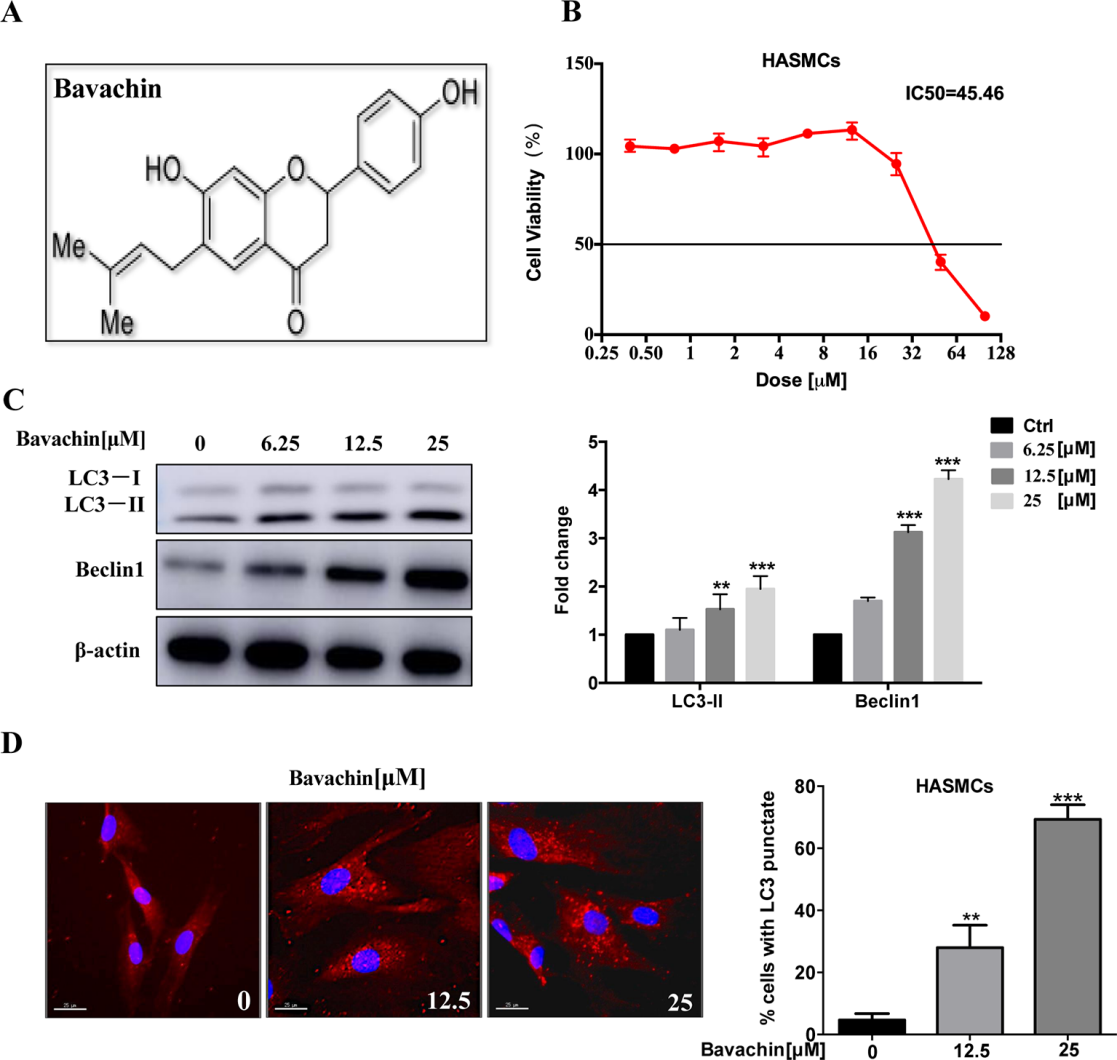
Induction of autophagy by bavachin in HASMCs. **(A)** Molecular structure of bavachin. **(B)** Cytotoxicity analysis of bavachin in HASMCs. **(C)** Western blot analysis of autophagy-related protein (LC3) expression in bavachin-treated HASMCs (**P < 0.01).The full-length images of Western blot are shown in **Figure S2C**. **(D)** Immunofluorescence analysis of LC3-II puncta formation (red color represents LC3-II, blue represents DAPI; scale bar = 25 μm). Statistical significance was analyzed by the t-test(**P < 0.01,***P < 0.001).The data is represented as mean ± S.D. (n = 3).

Previous studies have reported that bavachin has a variety of pharmacological effects, including anti-inflammatory, anti-oxidation, anti-ageing, anti-bacterial and estrogen-like effects, which molecular structure is shown in **Figure 2A**. *P. corylifolia* has been demonstrated to induce autophagy in PC‐3 cells (Lin et al., 2018). So, we will further study whether bavachin can activate autophagy in HASMCs. Therefore, we first examined the cytotoxicity of bavachin on HASMCs with various concentrations from 0 to 100 µM for 72 h using the MTT assay. The IC_50_ value of bavachin in HASMCs is 45.46 µM (**Figure 2B**). With the increasing concentrations of bavachin, the expression of autophagy-related proteins LC3-II was elevated in comparison with the control group (**Figure 2C**, *p < 0.01*). Of note, bavachin was observed to dose-dependently induce the autophagic puncta formation around the cell membrane of HASMCs (**Figure 2D**, *p < 0.01*).

### Bavachin Inhibits β-GP-Induced Calcification in HASMCs

**Figure 3:**
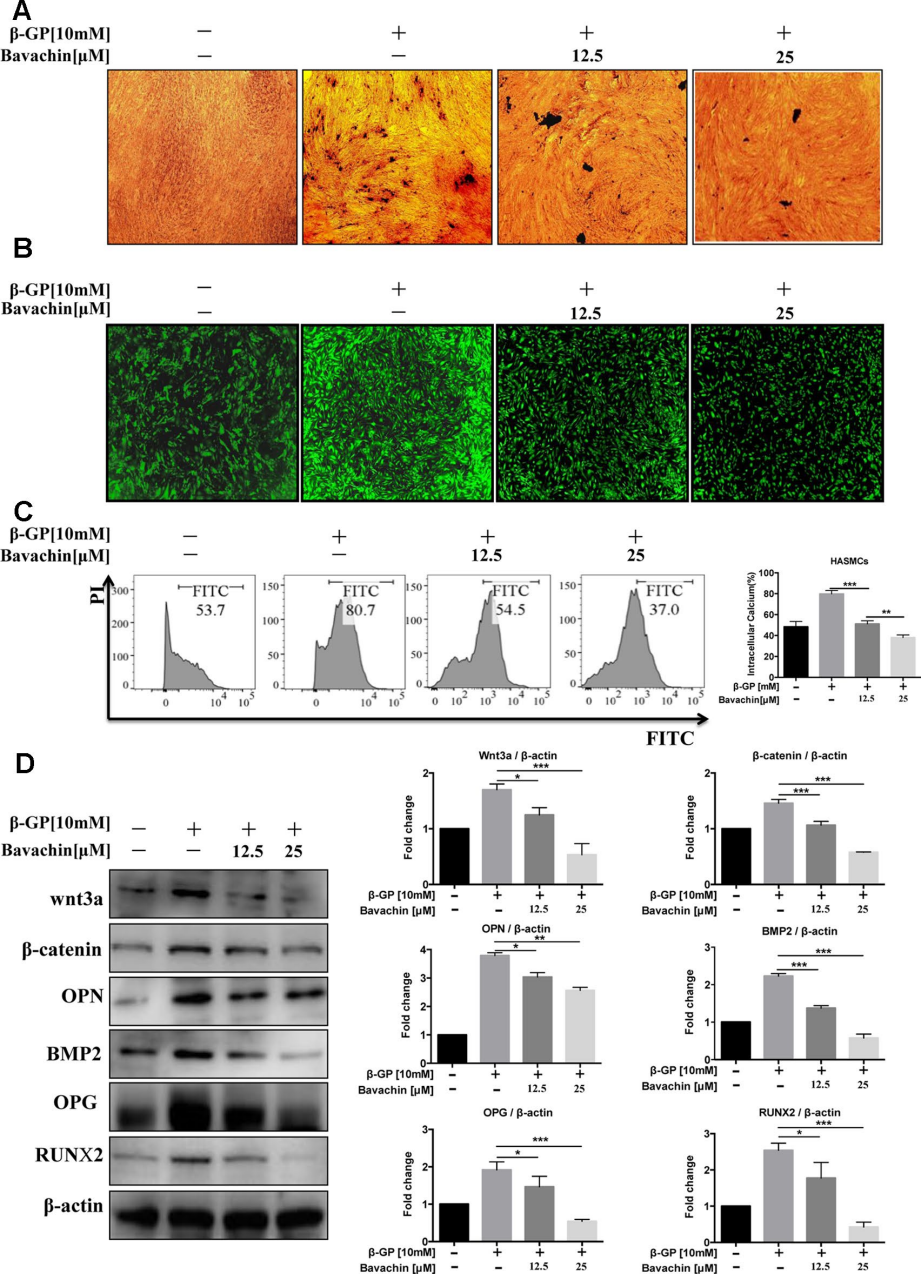
Bavachin inhibits β-GP-induced calcification in HASMCs. **(A)** Mineralization of calcium was quantified by VON KOSSA staining in bavachin and β-GP-treated HASMCs. The blackspots in the image are calcified deposition areas; magnification 10×. **(B)** Intracellular Ca^2+^was quantified by FLIPR calcium 6 dye (green) in bavachin- and β-GP-treated HASMCs; magnification 4×. **(C)** Flow cytometry analysis of intracellular Ca^2+^level in bavachin and β-GP-treated HASMCs. Statistical significance was analyzed by the t-test (**P < 0.01, ***P < 0.001). **(D)** Western blot analysis of calcification-related proteins (Wnt3a, β-catenin, OPN, BMP2, OPG, and Runx2) in HASMCs. The full-length images of Western blot are shown in **Figure S3D**. Statistical significance was analyzed by the one-way ANOVA (*P < 0.05, **P < 0.01, ***P < 0.001). The data is represented as mean ± S.D. (n = 3).

It is well known that β-GP (10 mM) increases the intracellular calcium level in HASMCs (Qiao et al., 2014). To investigate the effect of bavachin on the differentiation of HASMCs into osteoblasts, we cultured HASMCs with different concentrations of bavachin for 72 h. We then determined the impact of bavachin in reducing calcium level in β-GP-stimulated HAMSCs. With increasing concentrations of bavachin, VON KOSSA’s staining indicated that there were significantly less black-spots deposits in bavachin-treated HAMSCs (**Figure 3A**). Meanwhile, the intracellular calcium level was visualized in HASMCs loaded with FLIPR calcium 6 dye. The GFP-Ca^2+^ fluorescence signal increased significantly in β-GP-stimulated cells. However, the addition of bavachin was found to markedly suppress the β-GP-induced GFP-Ca^2+^ fluorescence in HASMCs (**Figure 3B**). Therefore, the level of intracellular calcium was gradually decreased in response to bavachin treatment (**Figure 3C**, *p < 0.01*).

β-GP was reported to stimulate the expression of osteoblast marker protein and up-regulation the Wnt/β-catenin signaling pathway in VSMCs (Cai et al., 2016). Of note, we found that the expression level of calcification-related proteins (Runx2, OPG, OPN, and BMP2) in bavachin-treated HASMCs were also reduced (**Figure 3D**, *p < 0.05*). Wnt/β-catenin signaling has been recognized as a major regulatory pathway for bone formation (Tu et al., 2015). Osteogenic genes, such as Runx2, is the main feature for osteogenic trans-differentiation and calcification in HASMCs induced under high-phosphate environment (Cencioni et al., 2013). Furthermore, several studies have shown that Wnt/β-catenin is a signal pathway closely related to vascular calcification (Albanese et al., 2018). Here, we demonstrated that the expression of β-catenin and Wnt3a, as well as the β-GP stimulated elevation of Runx2, OPG, OPN, BMP2 were significantly reduced (**Figure 3D**, *p < 0.05*). Collectively, our findings suggest that bavachin may reduce the β-GP-induced calcification of HASMCs *via* inhibition of the Wnt/β-catenin signaling.

### Bavachin Prevents β-GP-Induced Apoptosis in HASMCs

**Figure 4:**
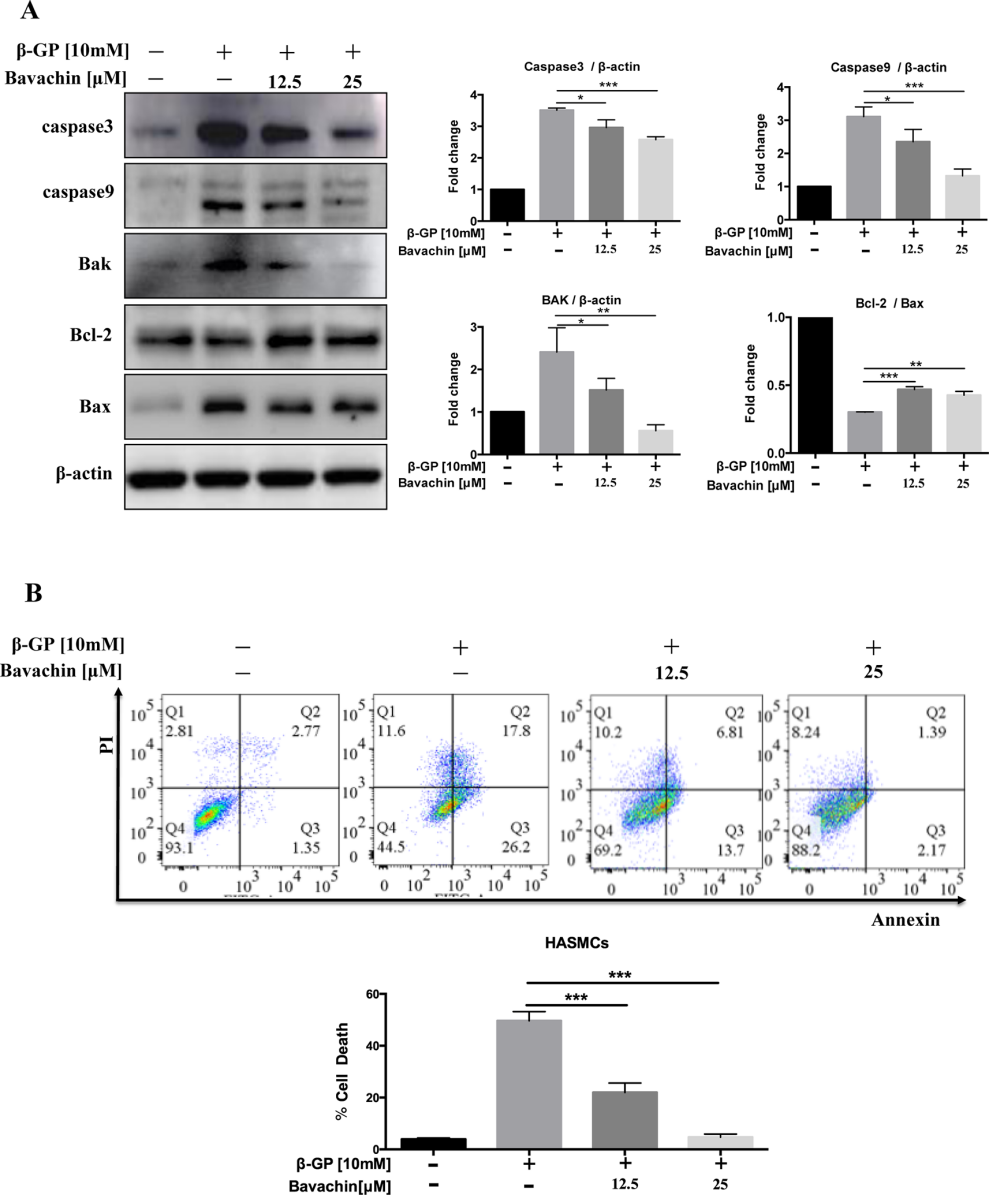
Bavachin suppresses β-GP-induced apoptosis in HASMCs. **(A)** Western blot analysis of apoptosis-related proteins (Caspase3, Caspase9, Bak, Bcl-2, and Bax) in bavachin and β-GP-treated HASMCs (^*^ *P < 0.05*, ^**^ *P < 0.01*, ^***^ *P < 0.001*). The full-length images of Western blot are shown in **Figure S4A**. **(B)** Flow cytometry analysis of apoptosis in bavachin and β-GP-treated HASMCs (^***^ *P < 0.001*). Statistical significance was analyzed by the one-way ANOVA (*P < 0.05, ^**^ *P < 0.01*, ^***^ *P < 0.001*). The data is represented as mean ± S.D. (n = 3).

Since β-GP can induce apoptosis in HASMCs (Qiu et al., 2017), to examine the downstream apoptotic signaling during β-GP-induced apoptosis in HASMCs, we examined the cleaved form of Caspase3 and Caspase9, Bak, and Bax of which the apoptosis-related protein, and Bcl-2, an inhibitory protein for apoptosis. Results indicated that bavachin dose-dependently suppressed the β-GP-induced expression of apoptosis-related proteins Bax, Bak, Caspase3 and Caspase9, it also prevented the β-GP-mediated suppression of Bcl-2, and the ratio of Bcl-2/Bax was gradually increased (**Figure 4A**, *p < 0.01*). On the other hand, Annexin V-PI flow cytometry analysis further confirmed the apoptosis induction by β-GP after 72 h treatment (**Figure 4B**, *p < 0.001*). Consistently, the addition of bavachin markedly prevented the cell death population in β-GP-treated HASMCs (**Figure 4B**, *p* < 0.001). It was demonstrated that bavachin could protect against β-GP-induced apoptosis in HASMCs.

### Bavachin Induces Autophagy *via* Inhibition of mTOR Signaling in HASMCs

**Figure 5:**
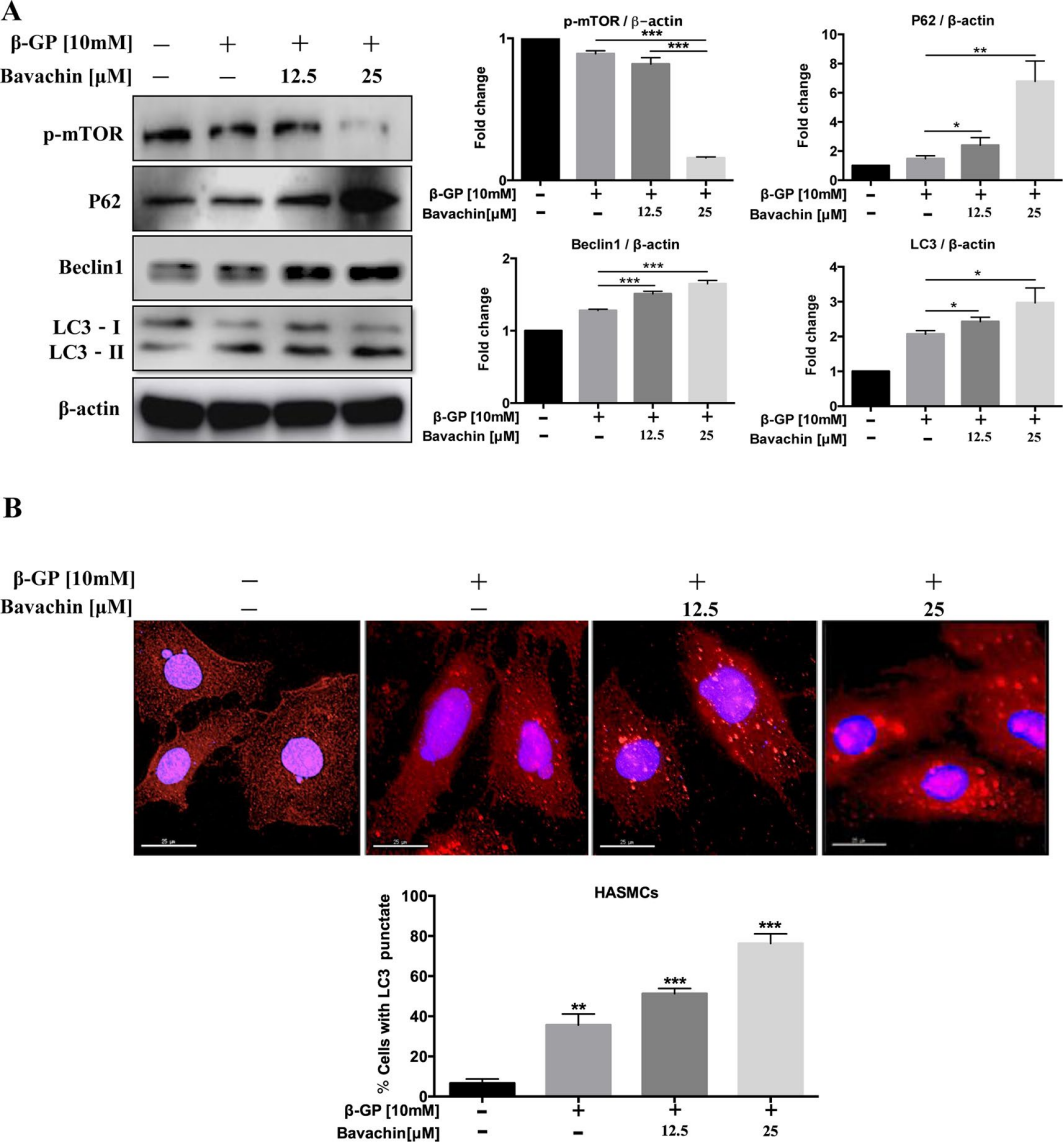
Bavachin activates autophagy via mTOR signaling in HASMCs. **(A)** Western blot analysis of mTOR and autophagy-related proteins (p62, beclin1, and LC3) in bavachin and β-GP-treated HASMCs. The full-length images of Western blot are shown in **Figure S5A**. **(B)** Immunofluorescence analysis of LC3-II puncta formation (red color represents LC3-II, blue represents DAPI; scale bar = 25 μm). Statistical significance was analyzed by the one-way ANOVA (*P < 0.05, **P < 0.01, ***P < 0.001). The data is represented as mean ± S.D. (n = 3).

Autophagy and apoptosis usually occur in the same cell, mainly in the sequence of autophagy before apoptosis (Marino et al., 2014). To determine whether autophagy could also be induced by bavachin after β-GP treatment in HASMC, we investigated the ratio of LC3-II/β-actin, beclin1, p62, and p-mTOR. Compared with the DMSO-treated control, we found that bavachin could also increase the ratio of LC3-II/β-actin, beclin1 and p62 in a dose-dependent manner; at the same time, the p-mTOR expression had an opposite trend (**Figure 5A**, *p < 0.05*). Besides, the immunofluorescence assay further revealed that β-GP alone would increase the intracellular localization of red LC3 puncta formation in comparison to control cells. Of note, bavachin treatment even showed more LC3 puncta formation on top of β-GP-stimulated cells (**Figure 5B**, *p < 0.01*).

### Bavachin Induces Phenotypic Switching in β-GP-Treated HASMCs

**Figure 6:**
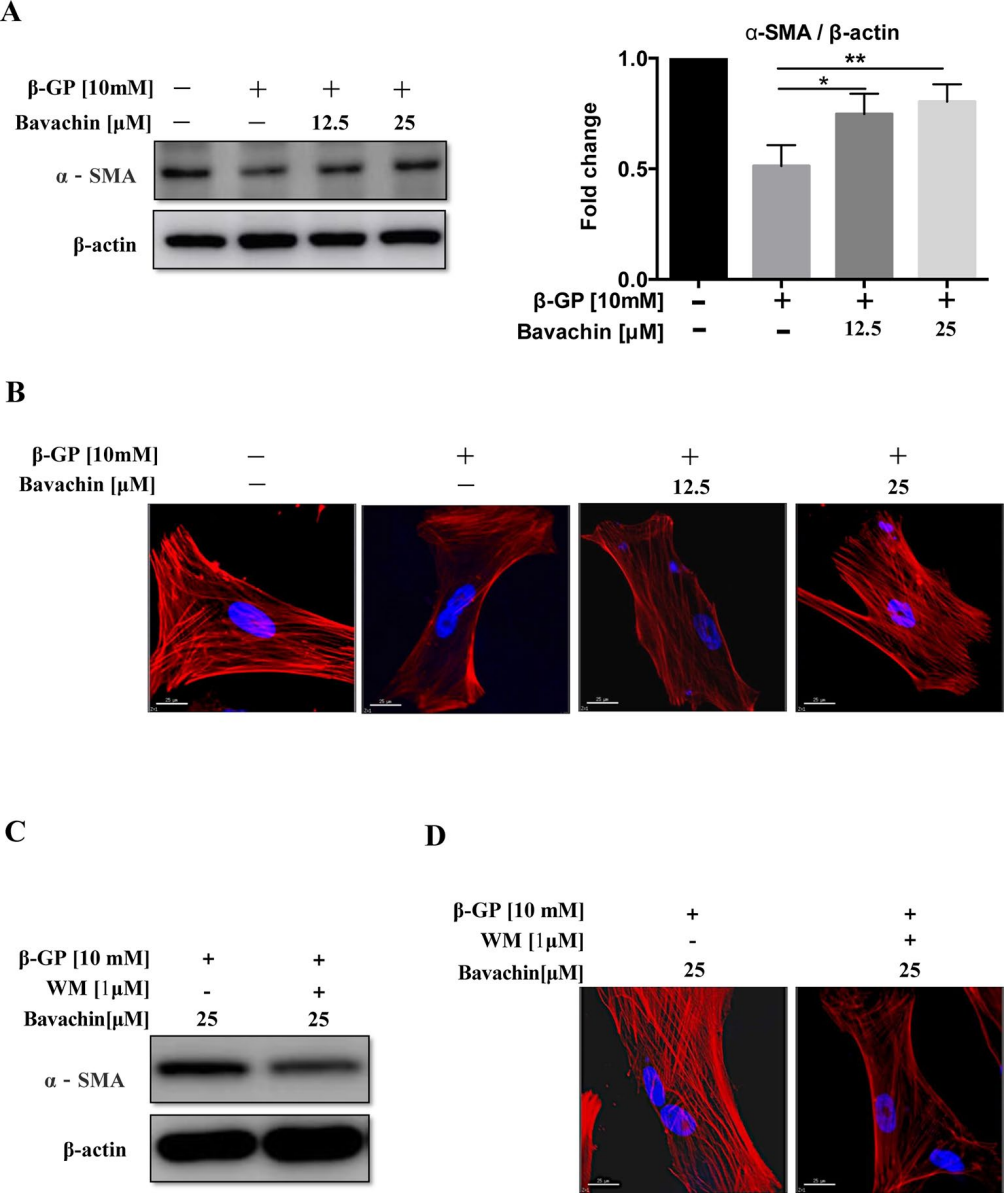
Autophagy participates the process of phenotypic switching in HASMCs. **(A)** Western blot detection of α-SMA (the smooth muscle cell phenotype protein), (*P < 0.05; **P < *0.01*). The full-length images of Western blot are shown in **Figure S6A**. **(B)** Observation of the smooth muscle cell cytoskeleton in HASMCs after treatment with β-GP and bavachin (red represents F-actin, blue represents DAPI; scale bar = 25 μm). **(C)** Western blot analysis showing levels of α-SMA and F-actin after administration of WM (1 µM) in HASMCs. The full-length images of Western blot are shown in **Figure S6C**. **(D)** Cytoskeletal staining assay verify osteoblast phenotype transformation (red represents F-actin, blue represents DAPI; scale bar = 25 μm). Statistical significance was analyzed by the one-way ANOVA (**P < 0.01). The data is represented as mean ± S.D. (n = 3).

The mechanism of VC formation is similar to that of bone formation. For example, the phenotype of VSMCs differentiates into osteoblast-like cells, thus forming osteoblast-like cells with different phenotypes (Giachelli, 2004). By analyzing the level of α-SMA (smooth muscle cell protein), we found that β-GP could reduce the expression level of α-SMA. As shown in (**Figure 6A**, *p < 0.01*) bavachin dose-dependently increased the expression of α-SMA in β-GP-treated HASMCs. By examining the smooth muscle cytoskeleton, results showed that there was a strong expression of F-actin in the untreated cells, whereas β-GP was shown to inhibit the expression and to ruin the cytoskeletal structure of F-actin in HASMCs (**Figure 6B**). However, the addition of bavachin abolished the damaging effect of β-GP and recovered the cytoskeletal structure of F-actin in HASMCs (**Figure 6B**). Collectively, the phenotypic transition process of SMCs to osteoblasts was, therefore inhibited.

To investigate whether bavachin-induced autophagy participates in the phenotypic transition process of SMCs. We incubated 25 µM of bavachin in HASMCs in the presence or absence of autophagy inhibitor WM (1 µM). Results demonstrated that blockage of bavachin-induced autophagy would eventually suppress the expression of α-SMA (**Figure 6C**). At the same time, the bavachin-treated cells incubated with WM demonstrated significantly lower expression of F-actin in HASMCs (**Figure 6D**), suggesting that bavachin-induced autophagy could prevent HASMCs from differentiating into osteoblasts.

### Bavachin Suppresses β-GP-Induced Calcification and Apoptosis *via* Induction of Autophagy

**Figure 7:**
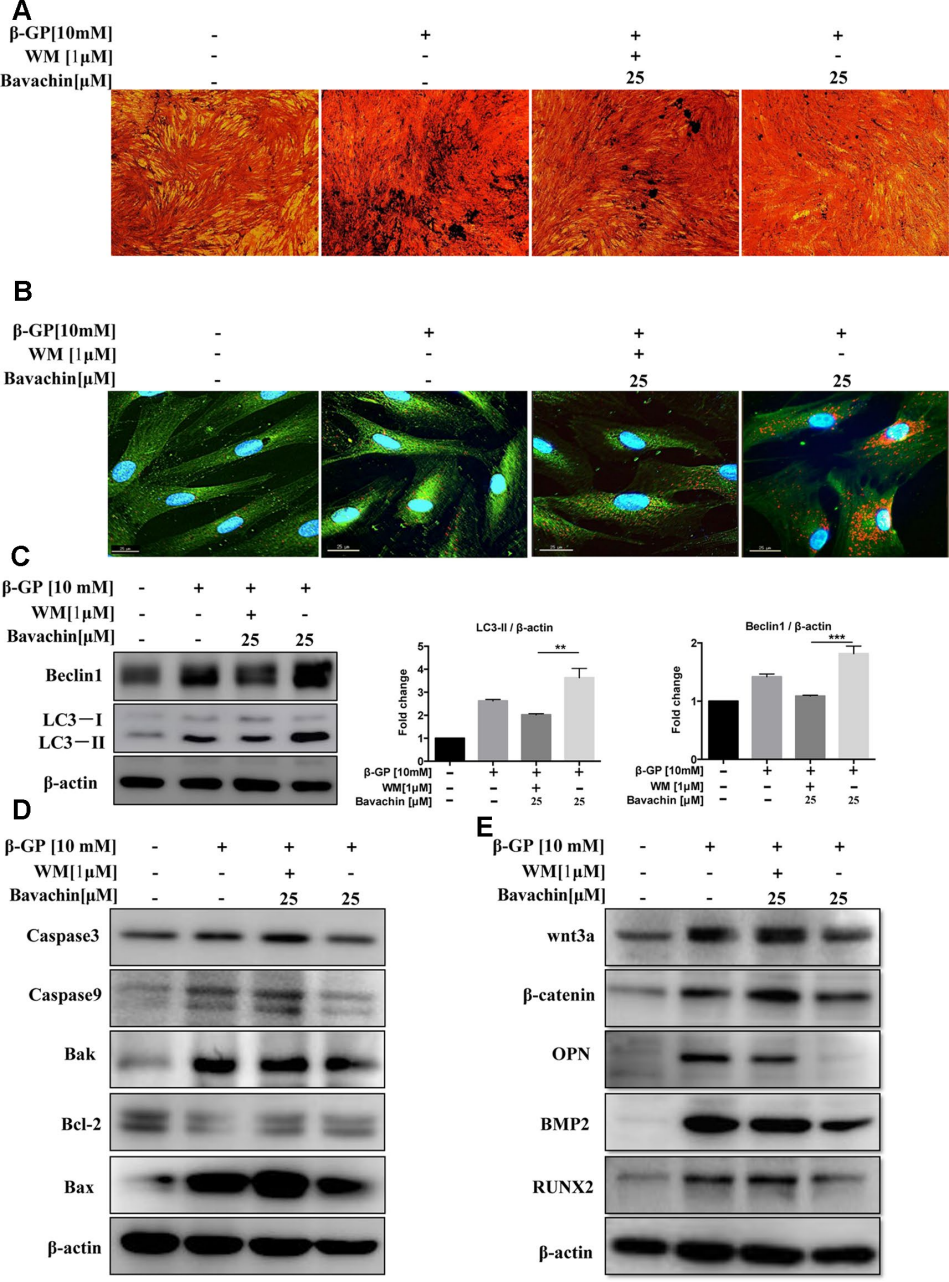
Bavachin suppresses β-GP-induced calcification and apoptosis *via* induction of autophagy. Effects of autophagy inhibitor WM in β-GP/bavachin-treated HASMCs. **(A)** A representative plate view of VONKOSSA staining was shown after application of WM in β-GP/bavachin-treated HASMCs. The black spots were observed in cytoplasm and nuclear regions of cells. **(B)** Immunofluorescence staining of calcification and autophagy-related proteins. Green GFP signal represents the calcified protein OPN, whereas red TRITC signal represents the autophagy protein LC3, and blue DAPI signal represents the nuclear region (scalebar = 25 μm). **(C)** Western blot analysis of autophagy proteins (LC3 and beclin1) in HASMCs treated with 10 mM β-GP and 25 μM bavachin for 72 h (**P < 0.01, ***P < 0.001). The full-length images of Western blot are shown in **Figure S7C**. **(D)** Western blot analysis of apoptotic proteins (Caspase3, Caspase9, Bak, Bcl-2, and Bax). The full-length images of Western blot are shown in **Figures S1A, S7D**. **(E)** Western blot analysis of calcification-related proteins: Wnt3a, β-catenin, OPN, BMP2, and Runx2. The full-length images of Western blot are shown in **Figures 1B**, **S7E**. Statistical significance was analyzed by the one-way ANOVA (**P < 0.01, ***P < 0.001). The data is represented as mean ± S.D. (n = 3).

To investigate whether bavachin-mediated autophagy induction is necessary for the prevention of β-GP-induced calcification and cell death in HASMCs. We therefore blocked the autophagic activity of bavachin by using WM. As shown in (**Figure 7A**), the β-GP-induced calcification in HASMCs was significantly reduced by bavachin, however, the addition of WM attenuated the inhibitory effect of bavachin and thereby resumed the β-GP-induced calcification. A representative plate view of the VON KOSSA staining demonstrated that administration of WM in β-GP/bavachin-treated cells displayed the increase of black spots in both cytoplasm and nuclear regions (**Figure 7A**). Concomitantly, immunofluorescence assay also verified the loss of autophagy effect in the presence of WM. As shown in (**Figure 7B**), the calcification-related protein OPN (green fluorescence) was significantly increased in β-GP-treated cells, whereas addition of bavachin markedly enhanced the puncta formed of autophagosome (red fluorescence) and reduced the expression signal of OPN. When the autophagy inhibitor, WM, was administrated in the calcification model, green fluorescence increased, and red fluorescence decreased, demonstrating that the calcification-related protein OPN increased, thus indicating that the calcification of HASMCs was enhanced when the autophagy activity was blocked (**Figure 7B**). Consistently, the addition of WM also suppressed the expression of autophagic marker protein LC3-II and Beclin1, which proteins were highly up-regulated in β-GP/bavachin-treated cells (**Figure 7C**, *p < 0.01*). Meanwhile, the expression of apoptosis-related proteins including Caspase3, Caspase9, Bak and Bax were increased significantly, while the anti-apoptotic protein, Bcl-2 was decreased in β-GP/bavachin-treated cells in response to the treatment of WM (**Figure 7D**), suggesting that blockage of autophagy would prompt to cell death in calcification model. Besides, blockage of autophagy by WM would further up-regulate the expression of calcification-related protein such as OPN, BMP2, and Runx2, as well as the appearance of β-catenin and Wnt3a (**Figure 7E**). Thus, current findings suggested that inhibition of autophagy can promote apoptosis and calcification *via* activation of Wnt/β-catenin signaling pathways. Inhibition of autophagy has been demonstrated to promote calcification of VSMCs (Durham et al., 2018). Hence, bavachin was verified to reduce β-GP-mediated calcification in HASMCs *via* induction of autophagy.

### Autophagy-Related Gene (Atg7) Is Required for Bavachin-Mediated Autophagy Induction and Prevention of Calcification and Apoptosis in β-GP-Treated HASMCs

**Figure 8:**
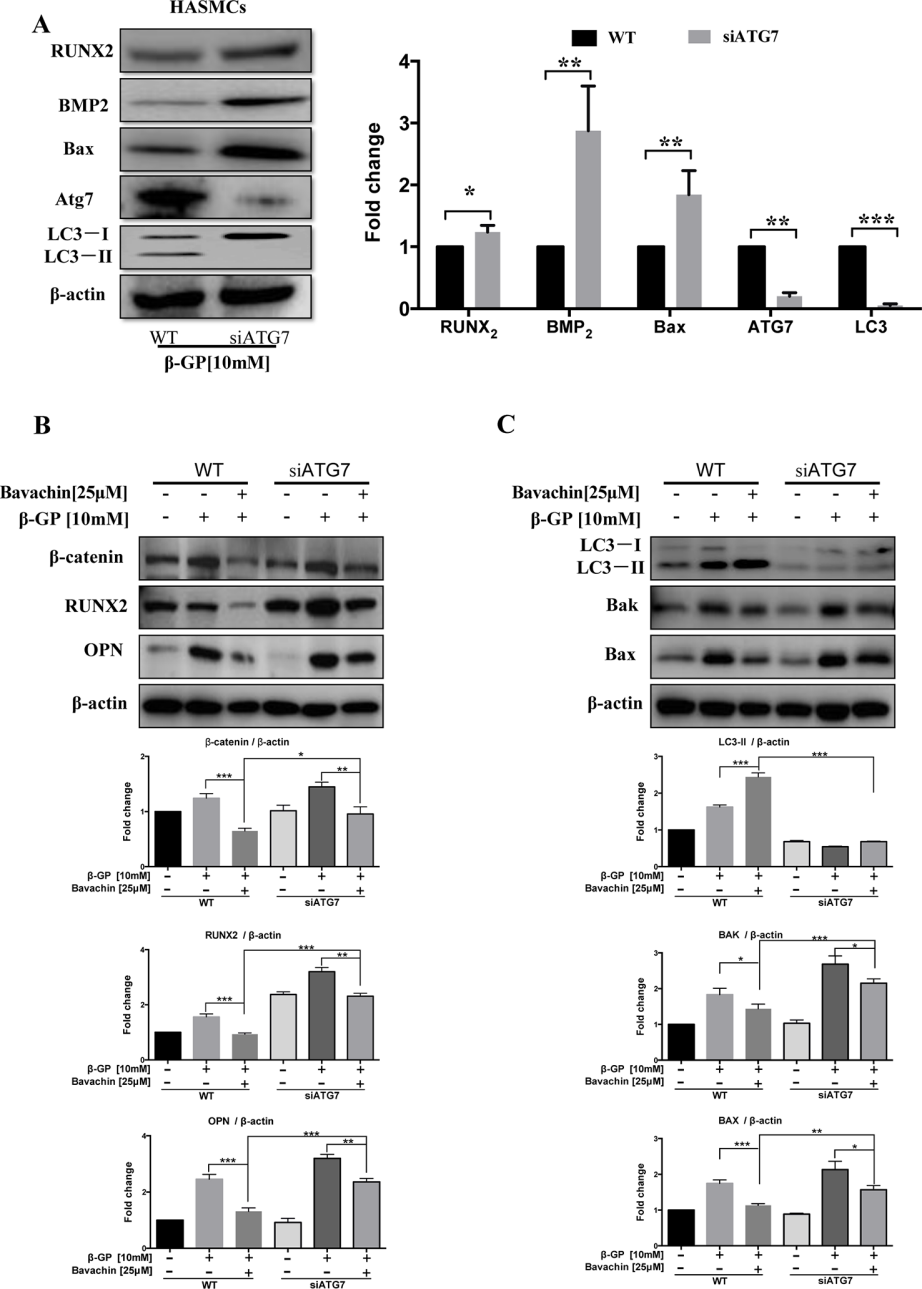
Bavachin inhibits calcification and cell apoptosis in β-GP-treated HASMCs *via* Atg7-dependent autophagy. **(A)** Protein expression difference in Atg7 knockdown HASMCs. Western blot analysis of apoptosis-related proteins (Bax) and calcification-related protein (Runx2 and BMP2) in β-GP treated HASMCs transfected with or without Atg7 siRNA. (*P < 0.05, **P < 0.01, ***P < 0.001). The full-length images of Western blot are shown in **Figure S8A**. **(B)** Western blot analysis of calcification-related proteins (β-catenin, Runx2, and OPN) in bavachin and β-GP-treated HASMCs transfected with or without Atg7 siRNA. The full-length images of Western blot are shown in **Figure S8B**. **(C)** Western blot analysis of apoptosis-related proteins (Bax and Bak) and LC3-II conversion in bavachin and β-GP-treated HASMCs transfected with or without Atg7 siRNA. The full-length images of Western blot are shown in **Figure S8C**. Statistical significance was analyzed by the one-way ANOVA (*P < 0.05, **P < 0.01, ***P < 0.001). The data is represented as mean ± S.D. (n = 3).

Atg7 is the upstream autophagy-related gene of LC3, and it is necessary to promote the conversion of LC3-I to LC3-II during the process of autophagy induction(Deshpande et al., 2018). Atg7 forms intracellular complexes with LC3 and is sensitive to thiol-oxidation. The interaction between Atg3 and Atg7 are required for membrane transfer and lipidation of LC3, which could be suppressed by enzymatic oxidation (Kaiser et al., 2012). Accordingly, we further determined the role of Atg7 in the autophagy. As shown in **Figure 8A**, knockdown of Atg7 in HASMCs eventually abolished the transform of LC3-I to LC3-II, indicating that Successful establishment of Atg7 knockdown cell model. We then verified the β-GP-induced expression of apoptosis-related proteins Bax. Results showed that there was an increase in expression of Bax in Atg7 knockdown HASMCs, demonstrating that inhibition of autophagy may enhance apoptosis in HASMCs, which findings coincided with the results of using WM in **Figure 7D**. For the expression of calcification-related proteins, OPN, Runx2, and BMP2 were all up-regulated in Atg7 knockdown HASMCs (**Figures 8A, B**) or the cells treated with WM (**Figure 7E**). To further demonstrate the role of Atg7 in apoptosis and calcification of HASMCs, we administered bavachin and β-GP in normal cells and Atg7 knockdown cells. Results indicated that both Bax and Bak expression were significantly increased in Atg7 knockdown cells, whereas the expression of calcification-related proteins such as β-catenin, Runx2, and OPN were all up-regulated (**Figures 8B, C**). Taken together, our findings revealed that bavachin requires Atg7 to induce autophagy, prevent β-GP-mediated apoptosis and calcification in HASMCs.

**Figure 9:**
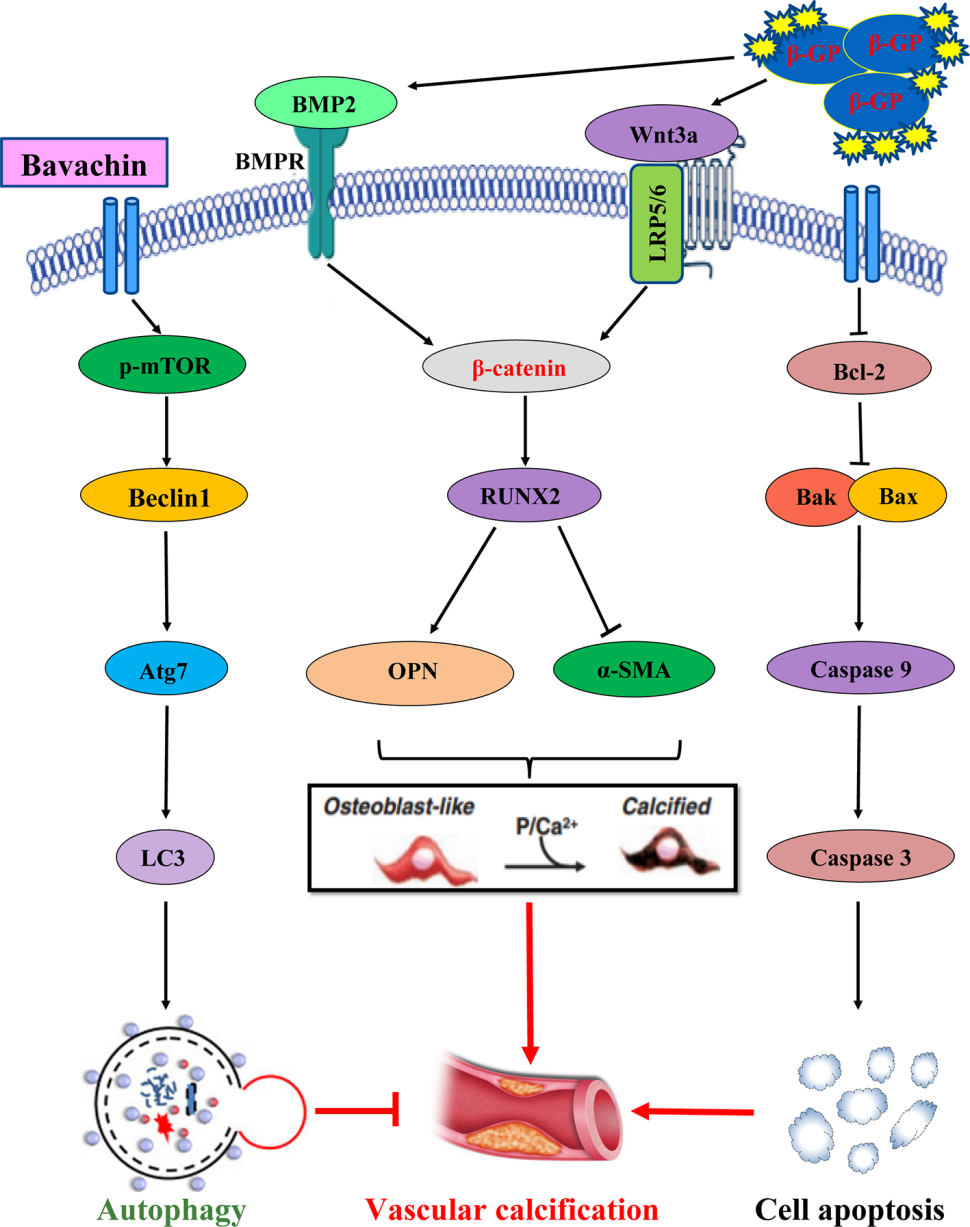
The possible mechanism by which Bavachin protects HASMCs against β-glycerophosphate-mediated VC and apoptosis, occurs via the activation of mTOR-dependent autophagy and suppression of β-catenin signalling.

As shown in **Figure 9**, the possible mechanism by which Bavachin protects HASMCs against β-glycerophosphate-mediated VC and apoptosis, occurs via the activation of mTOR-dependent autophagy and suppression of β-catenin signalling.

## Discussion

In this study, we investigated the effect of bavachin on the β-GP-induced apoptosis and calcification in HASMCs. The results showed that bavachin could inhibit β-GP-induced apoptosis, calcification, and activation of Wnt/β-catenin signaling in HASMCs. Also, bavachin activated autophagy, which could be attenuated by autophagy inhibition and Atg7 siRNA knockdown. Therefore, we concluded that bavachin protected HASMCs against apoptosis and calcification by activating the Atg7/mTOR‐dependent autophagy pathway and downregulating the Wnt/β-catenin signaling. Therefore, autophagy-activated drugs may be used as new drugs to prevent or treat vascular calcification, which provides a therapeutic strategy in the attenuation of vascular calcification.

As shown in **Figure 1**, β-GP not only stimulates calcification but also induces autophagy in HASMCs. In line with our result, Zhan et al., also demonstrated that β-GP significantly increased the calcification in VSMCs *simultaneously with the* activation of mTOR signaling (Zhan et al., 2014). They further clarified that the activation of autophagy by rapamycin inhibited β-GP-induced calcification (Zhan et al., 2014). Interestingly, another experiment showed that the induction of autophagy by β-GP is an adaptive response in the process of calcification, the inhibition of β-GP-mediated autophagy by either 3-methyladenine (3-MA) or siRNA approaches could exacerbate the calcification level in VSMCs. Conversely, activation of autophagy by valproic acid could protect VSMCs against β-GP-induced calcification (Shanahan, 2013). These findings further support our results that bavachin could suppress β-GP-induced calcification in HASMCs *via* induction of autophagy.

*In vivo* and *in vitro* evidence have shown that apoptosis is one of the mechanisms leading to vascular calcification, and a large number of researches have confirmed that apoptosis as one of the mechanisms of vascular calcification (Leopold, 2015). This also proves that there may be a link between calcification and apoptosis. The cysteine proteases Caspase3 and Caspase9 are considered to be important in the process of apoptosis, and their expression can be investigated when studying apoptosis and necrosis. In addition, Bcl-2, a gene that inhibits apoptosis transmission can inhibit apoptosis and promote cell survival, while Bax and Bak are pro-apoptotic genes, and their secretion is positively correlated with apoptosis (Lu et al., 2016; Peng et al., 2016). Apoptosis of VSMCs was detected in human atherosclerotic plaque, which may be induced by the interaction between death ligand and receptor or oxidative stress after macrophage damage. In many experimental models of calcification, apoptosis has been shown to play a pivotal role. A recent study demonstrated that an increase of oxidative stress could induce cartilage endplate cell apoptosis and calcification (Zhang et al., 2019). Here, we found that β-GP not only induced calcification but also induced apoptosis in HASMCs. The expression of proteins involved in both calcification and apoptosis was consistent. In addition, we found that β-GP-induced expression of calcification proteins may be activated by the Wnt/β-catenin signaling pathway while inducing apoptosis. When bavachin was administered, we discovered that β-GP-induced calcification or apoptosis could be reduced as well. This finding indicated that β-GP induced apoptosis and calcification in HASMCs, both of which are concentration-dependent, while bavachin can reduce calcification and apoptosis by some pathways.

Autophagy and apoptosis are two important catabolic processes that help maintain cell and tissue homeostasis (Gudipaty et al., 2018). Autophagy controls the renewal of protein aggregates and damaged organelles within the cells, which is the primary mechanism by which unwanted cells are removed and eliminated from the organism (Metaxakis et al., 2018). Although there are significant differences between the two pathways, they are highly correlated in determining cell fate. In our experiments, we found that bavachin could activate autophagy by up-regulation of LC3-II conversion, Beclin‐1 and p62 expression, whereas p-mTOR was decreased after bavachin treatment, indicating that bavachin activated autophagy *via* the mTOR-dependent pathway in HASMCs. We also showed that β-GP-induced HASMC calcification and apoptosis was alleviated when the cells treated with autophagy enhancer, bavachin. With increasing concentrations of bavachin, the expression of calcification-related proteins was decreased, and we can conclude that autophagy plays a protective role in the apoptosis of HASMCs.

Autophagy has two possibilities for apoptosis, one is protective, and the other is damage. We found that bavachin can reduce β-GP-induced calcification by activating autophagy. To investigate the relationship between autophagy, calcification and apoptosis in HASMCs during calcification, treated with the autophagy inhibitor wortmannin (WM) which suppress the phosphoinositide 3-kinase (PI3K) pathway were found that bavachin‐induced autophagy was downregulated. Contrary, both apoptosis and VSMC-osteoblasts transition were enhanced after the administration of β-GP (**Figures 7D, E**). Our results suggested that bavachin‐induced autophagy can prevent apoptosis and VSMC-osteoblasts transition of HASMC cells

The Wnt/β-catenin signaling pathway is an important pathway for osteogenic differentiation. The high phosphate environment activates this signal transduction pathway, and the downstream signaling cascade after binding of Wnt to its receptor and transports β-catenin to the nucleus to upregulate Runx2 expression (Huang and He, 2008). OPG participates in the regulation of tissue calcification, control of bone resorption by inhibiting further differentiation of osteoblasts into osteoclasts and is highly expressed in osteoblasts. Previous studies have demonstrated that OPG is a crucial biomarker protein for atherosclerosis (AS) and medical artery calcification (MAC) (Krzanowski et al., 2018). In this study, when autophagy was activated, the downstream proteins BMP2, Runx2, OPG, and OPN were decreased together with β-catenin and Wnt3a. With WM treatment or knockdown of Atg7 in HASMCs, β-catenin and Wnt3a were all up-regulated.

Autophagy is involved in the process of phenotypic switching of HASMCs. Interestingly, autophagy not only induces phenotypic transformation of VSMC but also plays a key role in cellular stress response (Grootaert et al., 2018). By determining α-SMA levels (the smooth muscle cell phenotype protein), we found that β-GP could reduce α-SMA. When the cells were treated with bavachin, the suppression of α-SMA is alleviated, we discovered that phenotypic transformation of smooth muscle cells can be protected by activating autophagy. It was further demonstrated that bavachin-induced autophagy could protect HASMCs from calcification and apoptosis. In summary, bavachin can be used as an autophagy agonist to activate autophagy to protect against arteriosclerosis, and further research is needed.

Here, we concluded that bavachin suppresses apoptosis and calcification effects in HASMCs. The mechanism of action of the compound is dependent on Atg7/mTOR-mediated autophagy pathway and suppression of β-catenin signaling, thereby protects HASMCs against apoptosis and calcification

## Data Availability

The raw data supporting the conclusions of this manuscript will be made available by the authors, without undue reservation, to any qualified researcher. 

## Author Contributions

VW and Y-ZH conceived and designed the research. H-QH drafted the manuscript. NZ, C-lQ, Y-QQ, A-GW, YH, QS, W-LZ, and YL performed experiments. H-QH and Y-QQ analyzed data. H-QH, BL, and VW edited the article. VW and Y-ZH approved the final version of the article.

## Conflict of Interest

The authors declare that the research was conducted in the absence of any commercial or financial relationships that could be construed as a potential conflict of interest.

## Abbreviations

VC, Vascular calcification; TCM, Traditional Chinese medicines; β-GP, β-glycerophosphate; HASMCs, human aortic smooth muscle cells; OPG, osteoprotegerin; OPN, osteopontin; Runx2, runt‐related protein 2; BMP2, bone morphogenetic protein-2; Atg, Autophagy Related gene; WM, wortmannin; VSMCs, Vascular smooth muscle cells; m-TOR, mechanistic target of rapamycin; ECM, extracellular matrix; CD, cardiovascular diseases; AS, arteriosclerosis; MTT, 3-(4,5-dimethylthiazol-2-yl)-2,5-diphenyltetrazolium bromide; IC_50_, half maximal inhibitory concentration; LSD, Least Significant Difference; α-SMA, Alpha-smooth muscle actin; DMSO, Dimethyl sulfoxide; PI3K, phosphoinositide 3-kinase; MAC, medical artery calcification; LC3-II, LC3-phosphatidylethanolamine conjugate; Wnt, Wingless and the name Int-1; TRITC-LC3, Tetramethylrhodamine-LC3; PC-3 cells, Caspase3, containing asparte-specific protease 3; Caspase9, containing asparte-specific protease 9; Bcl-2, B-cell lymphoma 2; Bax, Bcl-2-associated X protein; Bak, Bcl-2 antagonist/killer protein; p-mTOR, Phospho mechanistic target of rapamycin; Beclin1, Autophagy Related gene 6; P62, Sequestosome-1.
