# Supplementary material for: Bavachin Protects Human Aortic Smooth Muscle Cells Against β-Glycerophosphate-Mediated Vascular Calcification and Apoptosis via Activation of mTOR-Dependent Autophagy and Suppression of β-Catenin Signaling
Source: Front Pharmacol. 2019 Dec 19;10:1427. doi: 10.3389/fphar.2019.01427 (PMC6930901; doi:10.3389/fphar.2019.01427)

Bavachin protects human aortic smooth muscle cells against β-glycerophosphate-mediated vascular calcification and apoptosis via activationof mTOR-dependent autophagy and suppression of Wnt signaling

Hu-Qiang He[1,2,3], Betty Yuen Kwan Law[1,2], Ni Zhang[1,2], Cong-ling Qiu[1,2], Yuan-Qing Qu[1,2], An-Guo Wu[6,7], Yu Han[1,2], Qi Song[1,2,8], Wen-Lu Zheng[1,2,4,5], Yong Liu[3], Yan-Zheng He[*3] and Vincent Kam Wai Wong[*1,2]

[1]Faculty of Chinese Medicine Macau University of Science and Technology MacauPeople's Republic of China

[2]State Key Laboratory of Quality Research in Chinese Medicine (Macau University of Science and Technology)MacauPeople's Republic of China

[3]Department of Vascular Surgery,Affiliated Hospital of Southwest Medical UniversityLuzhouPeople's Republic of China

[4]Department of Nuclear Medicine, Affiliated Hospital of Southwest Medical University, Luzhou, China

[5]Nuclear Medicine and Molecular Imaging Key Laboratory of Sichuan Province, Luzhou, China

[6]Laboratory of Chinese Materia Medical, Department of Pharmacology, School of Pharmacy, Southwest Medical University, Luzhou, Sichuan, China

[7]Institute of Cardiovascular Research, the Key Laboratory of Medical Electrophysiology, Ministry of Education of China, Collaborative Innovation Center for Prevention and Treatment of Cardiovascular Disease of Sichuan Province, Southwest Medical University, Luzhou, Sichuan, China;

[8]Department of Thoracic and Cardial surgery,Affiliated Hospital of Southwest Medical UniversityLuzhouPeople's Republic of China

*** Correspondence:**

Vincent KamWai Wong, Ph.D., Associated Professor, E-mail: kawwong@must.edu.mo;

Yan-zheng He, Ph.D., Professor, University president, E-mail:heyanzheng_2018@163.com;

1 Supplementary Figure

Figure1. β-GP induces calcification in HASMCs. (C) Expression of calcification-related proteins (OPG, RUNX2, OPN and BMP2) were analyzed by Western blotting. The full-length images of Western blot are shown in **Figure S1 C** (D) Expression of autophagy-related proteins (LC3-I/II and Beclin1) were analyzed by Western blotting. The full-length images of Western blot are shown in **Figure S1 D**

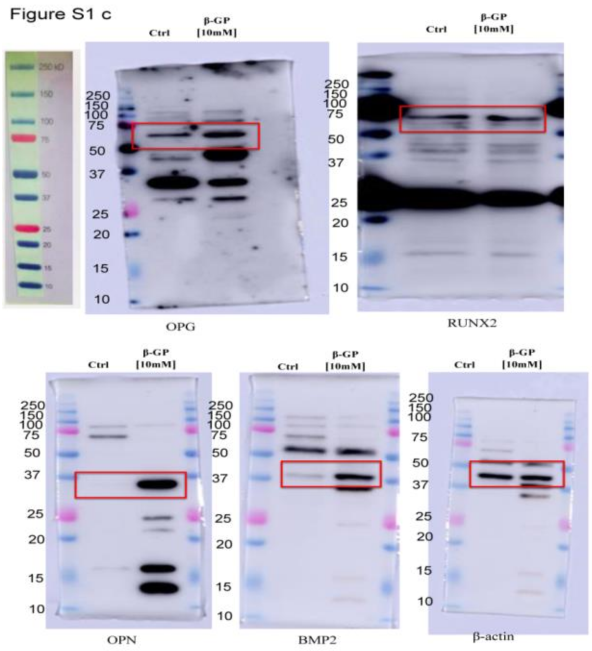

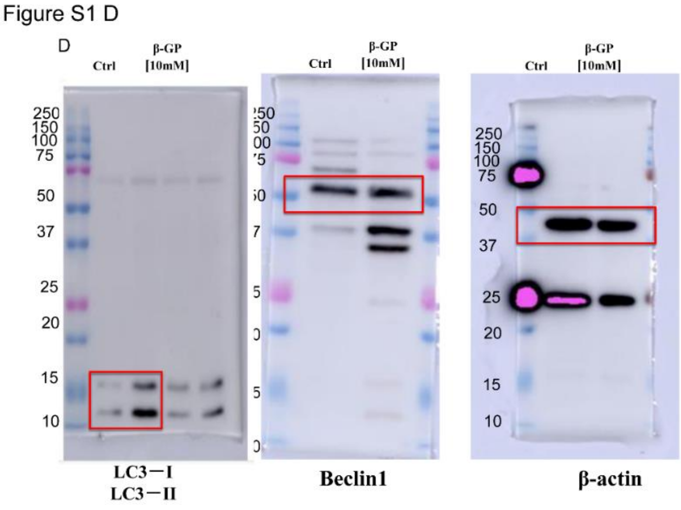

Figure2.Induction of autophagy by Bavachin in HASMCs. (C) Western blot analysis of autophagy-related protein (LC3) expression in Bavachin-treated HASMCs. The full-length images of Western blot are shown in **Figure S2 C**

Figure S2 C

C

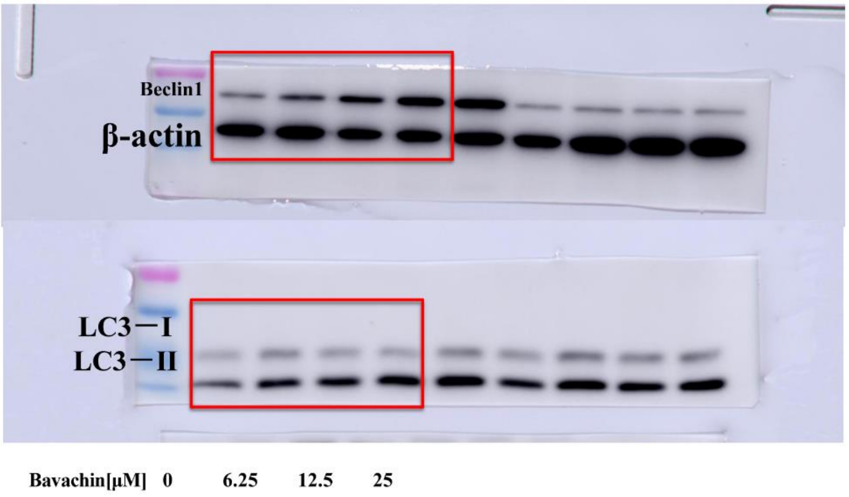

Figure3:Bavachin inhibits β-GP-induced calcification in HASMCs. (D) Western blot analysis of calcification-related proteins (Wnt3a, β-catenin, OPN,BMP2,OPG,and RUNX2) in HASMCs. The full-length images of Western blot are shown in **Figure S3 D**

Figure S3 D

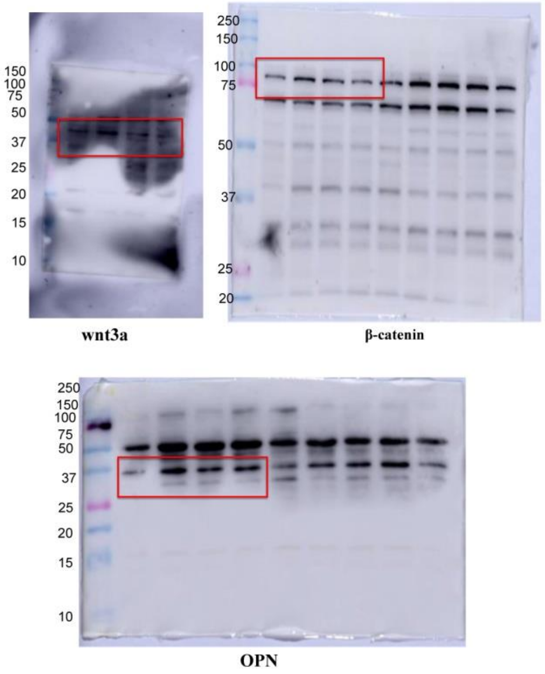

Figure s3 D

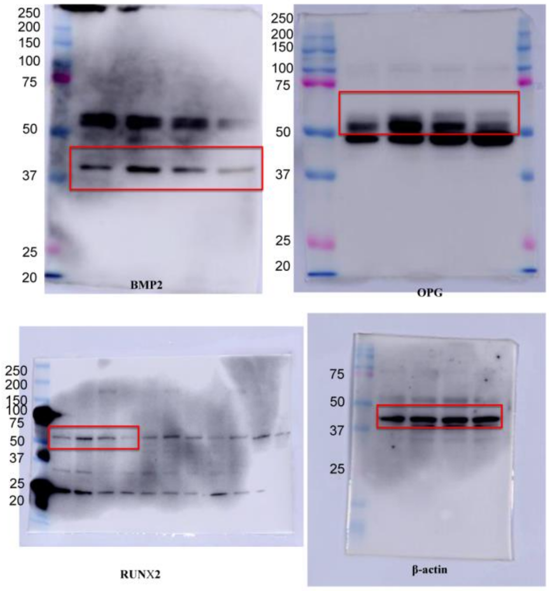

Figure4: Bavachinsuppressesβ-GP-induced apoptosis in HASMCs.(A) Western blot analysis of apoptosis-related proteins (caspase-3, caspase-9, Bak, Bcl-2, and Bax) in Bavachin- and β-GP-treatedHASMCs The full-length images of Western blot are shown in **Figure S4 A**

Figure S4 A

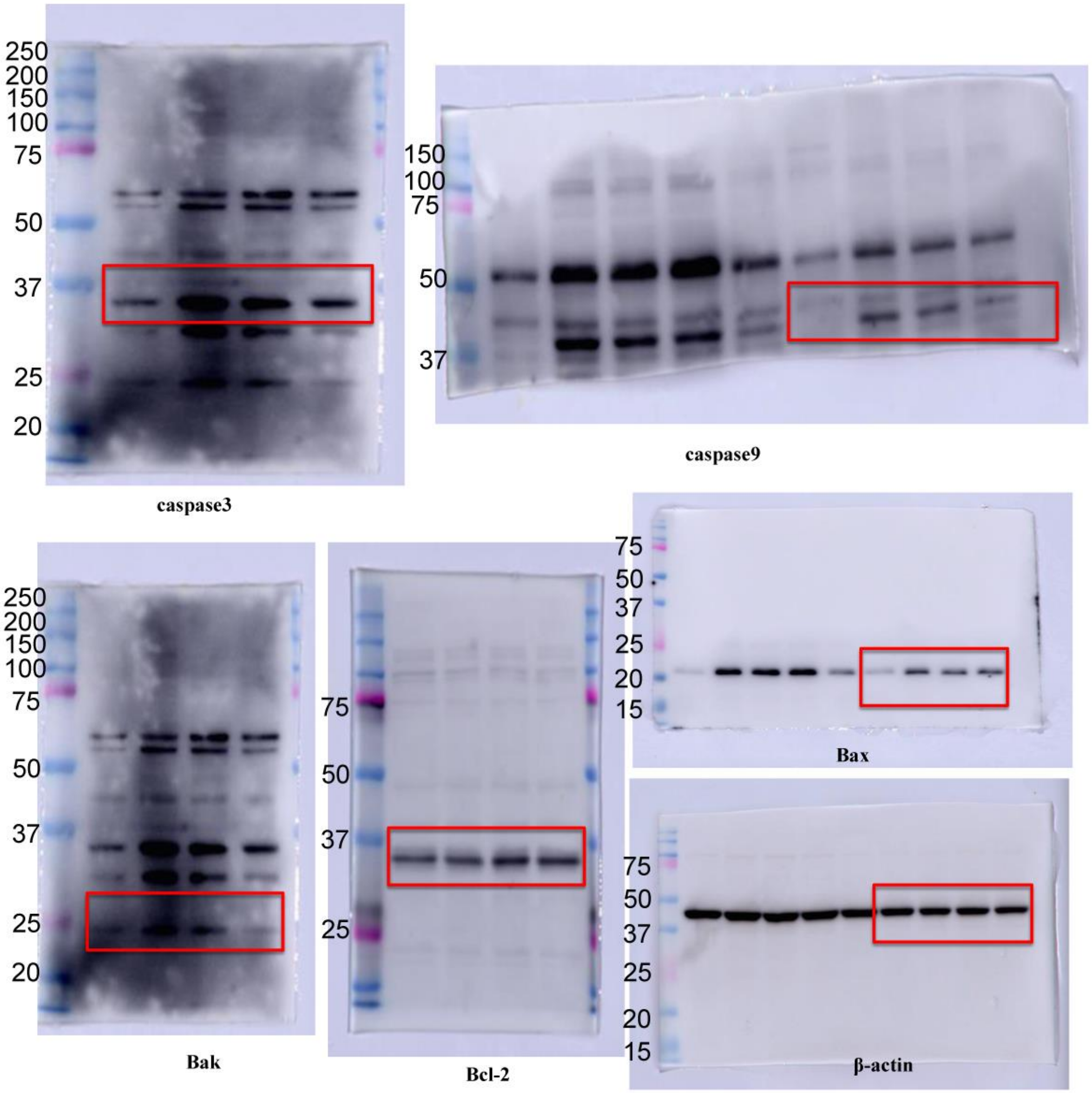

Figure5. Bavachin activates autophagy *via* mTOR signaling in HASMCs. (A) Western blot analysis of mTOR and autophagy-related proteins (p62, beclin1, and LC3) in Bavachin- and β-GP-treated HASMCs. The full-length images of Western blot are shown in **Figure S5A**

Figure S5 A

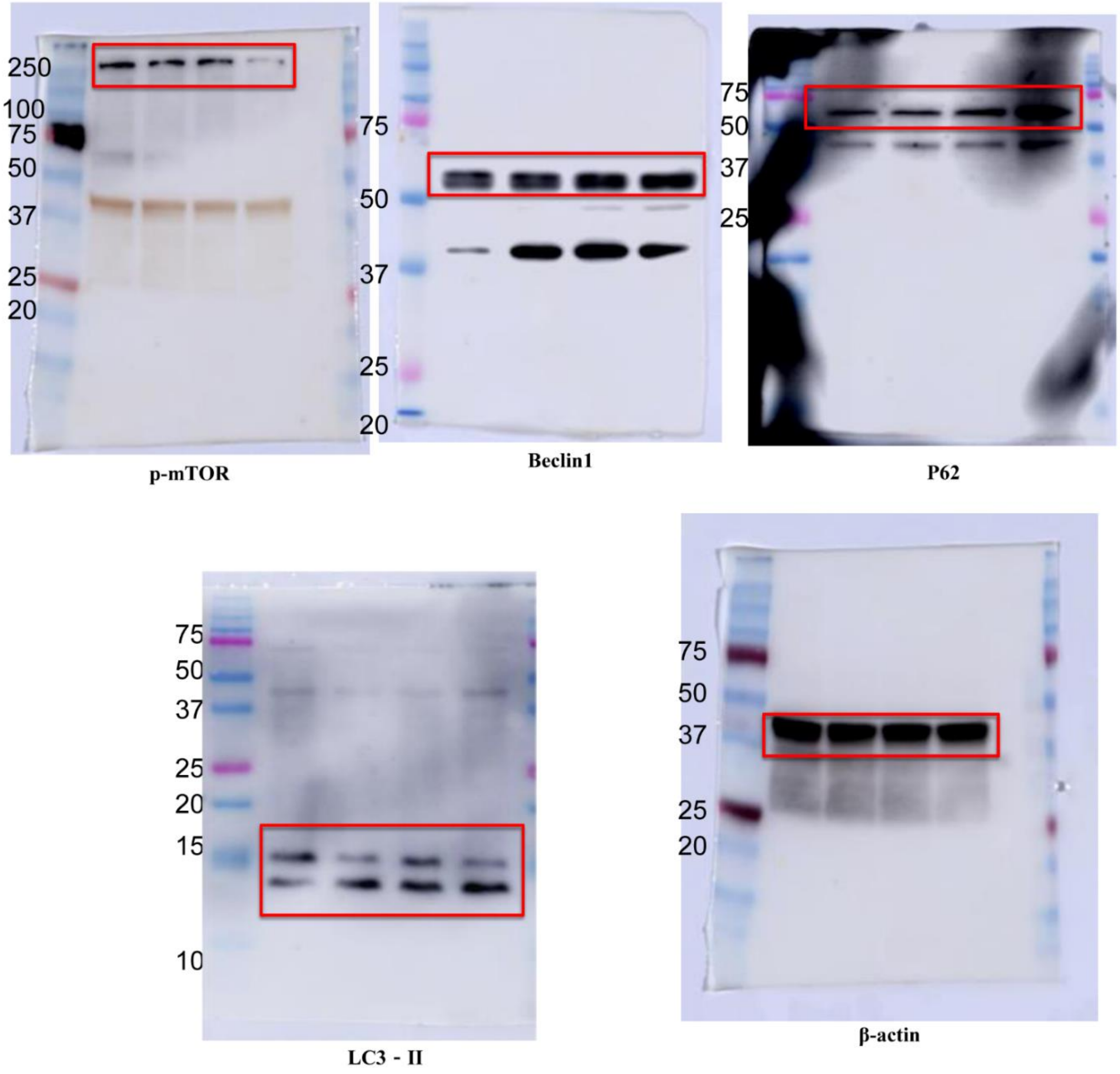

Figure 6:Autophagy participates the process of phenotypic switching in HASMCs.(A) Western blot detection of α-SMA (the smooth muscle cell phenotype protein), (***P*<0.01). The full-length images of Western blot are shown in **Figure S6 A** (C)Western blot analysis showing levels of α-SMA and F-actin after administration of wortmannin (WM)[1 μM] in HASMCs. The full-length images of Western blot are shown in **Figure S6 C.**

Figure s6 A

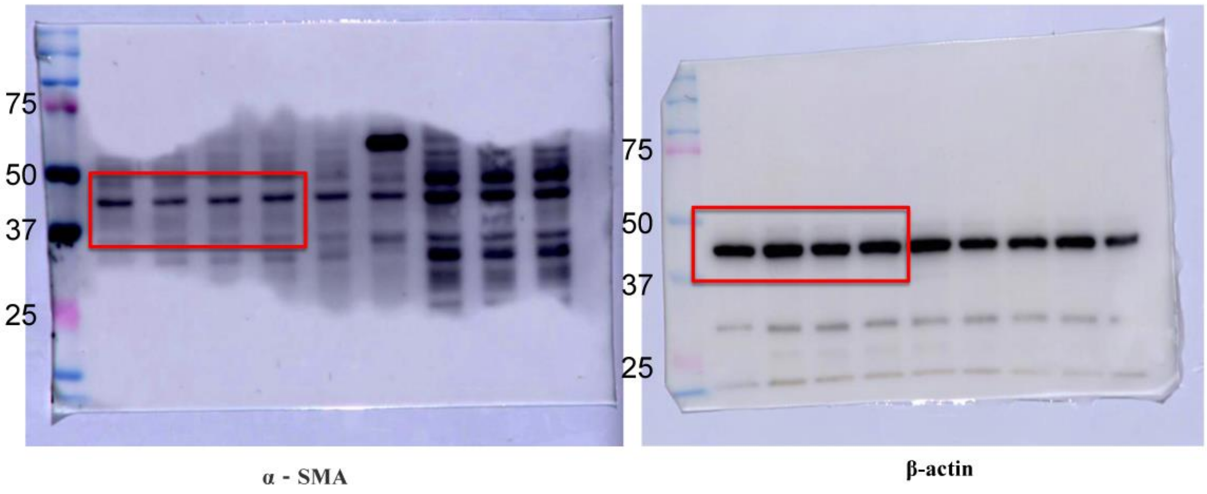

Figure s6 C

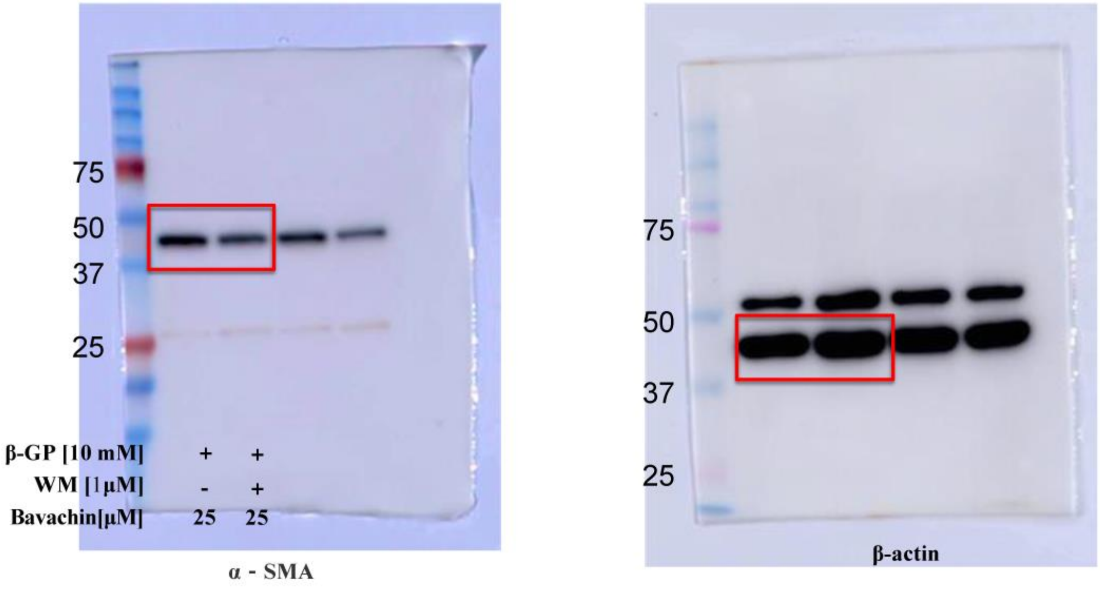

Figure 7. Bavachin suppresses β-GP-induced calcification and apoptosis *via* induction of autophagy. (C)Western blot analysis of autophagy proteins (LC3, beclin1)in HASMCs treated with 10 mM β -GP and 25 μM Bavachin for 72 h. (***P*<0.01****P*<0.001) The full-length images of Western blot are shown in **Figure S7 C** (D) Western blot analysis of apoptotic proteins(caspase-3, caspase-9,Bak,Bcl-2 andBax). The full-length images of Western blot are shown in **Figure S7 D.** (E) Western blot analysis of calcification-relatedproteins:Wnt3a, β-catenin,OPN,BMP2and RUNX2). The full-length images of Western blot are shown in **Figure S7 E**

Figure s7 C

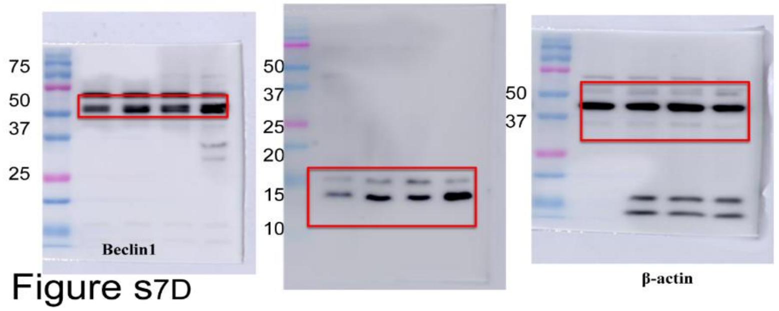

Figure s7D

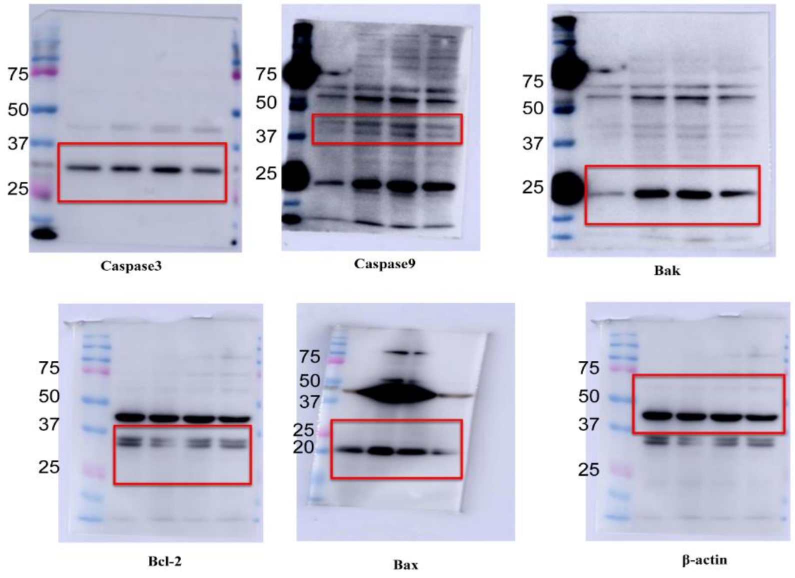

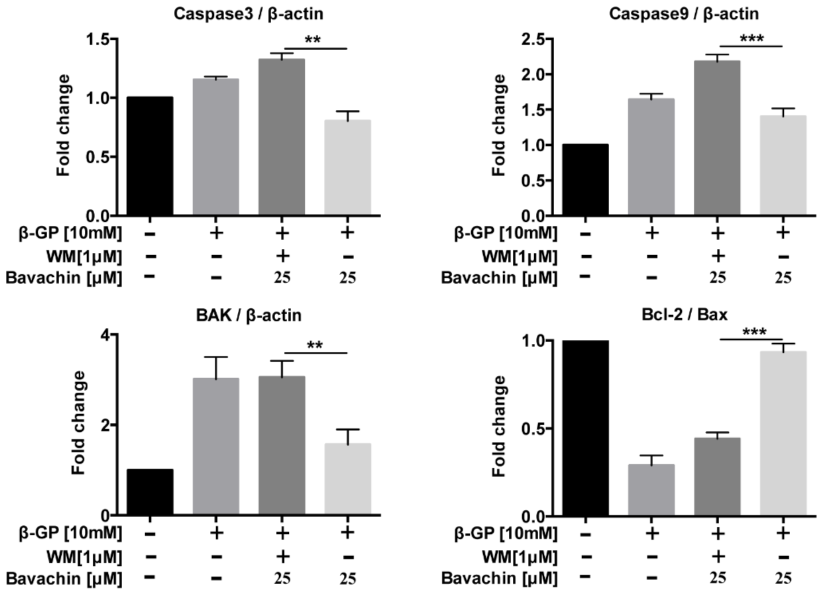

Figure s7 E

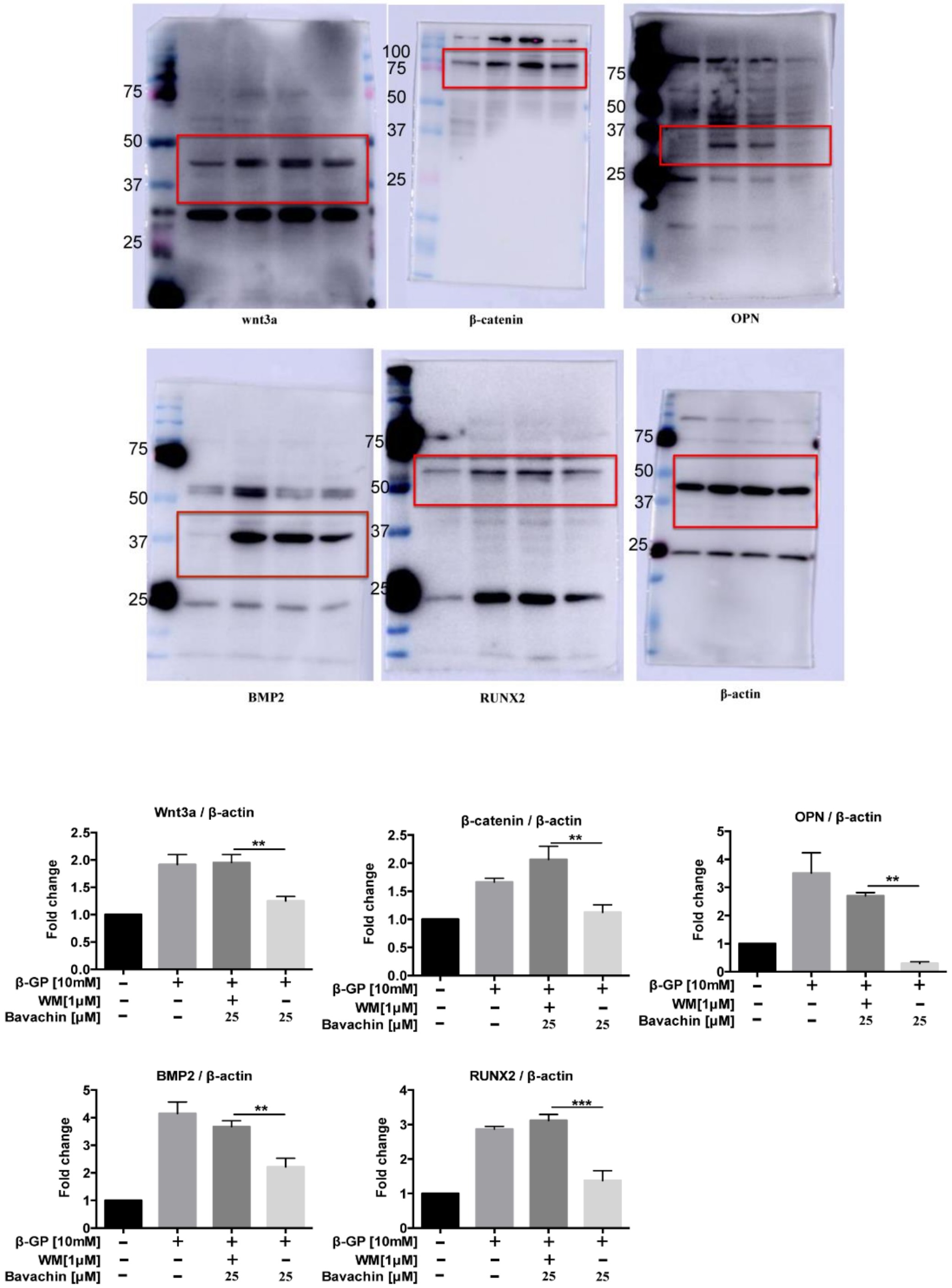
75
50
37
25
wnt3a
100
75
50
37
25
β-catenin
75
50
37
25
OPN
75
50
37
25
BMP2
75
50
37
25
RUNX2
75
50
37
25
β-actin
Wnt3a / β-actin
β-catenin / β-actin
OPN / β-actin
BMP2 / β-actin
RUNX2 / β-actin
Fold change
β-GP [10mM]
WM[1μM]
Bavachin [μM]
25
**
***

Figure8:Bavachin inhibits calcification and cell apoptosis in β-GP-treated HASMCs *via* ATG7-dependent autophagy. (A)Protein expression difference in Atg7 knockdown HASMCs.Western blot analysis of apoptosis-related proteins (Bax) and calcification-related protein (RUNX2 and BMP2) in β-GP treated HASMCs transfected with or without Atg7 siRNA. (*P<0.05,**P<0.01,***P<0.001) The full-length images of Western blot are shown in **Figure S8 A**(B) Western blot analysis of calcification-related proteins (β-catenin, RUNX2, and OPN) in Bavachin- and β-GP-treated HASMCs transfected with or without Atg7 siRNA. The full-length images of Western blot are shown in **Figure S8 B** (C)Western blot analysis of apoptosis-related proteins (Bax and Bak)and LC3-II conversion in Bavachin- and β-GP-treated HASMCs transfected with or without Atg7 siRNA. The full-length images of Western blot are shown in **Figure S8 C.**

Figure S8 A

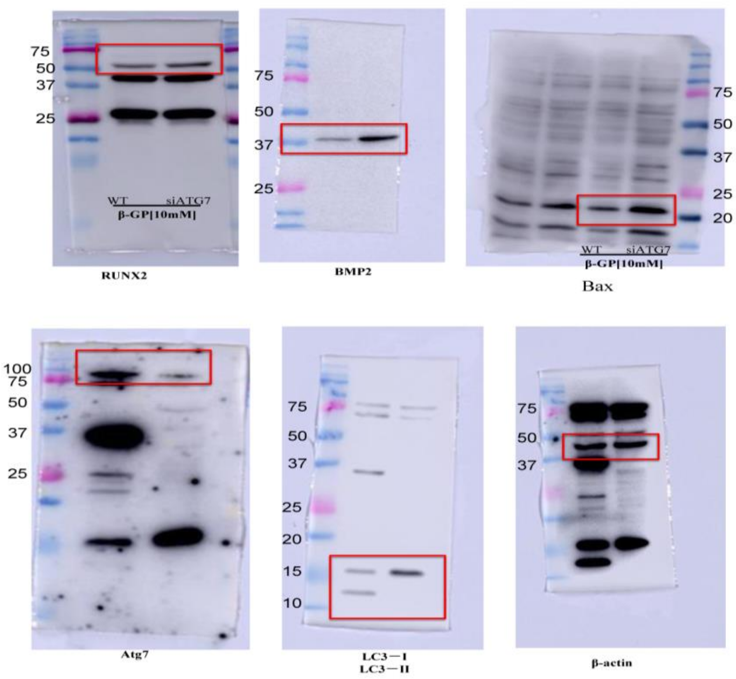

Figure S8 B

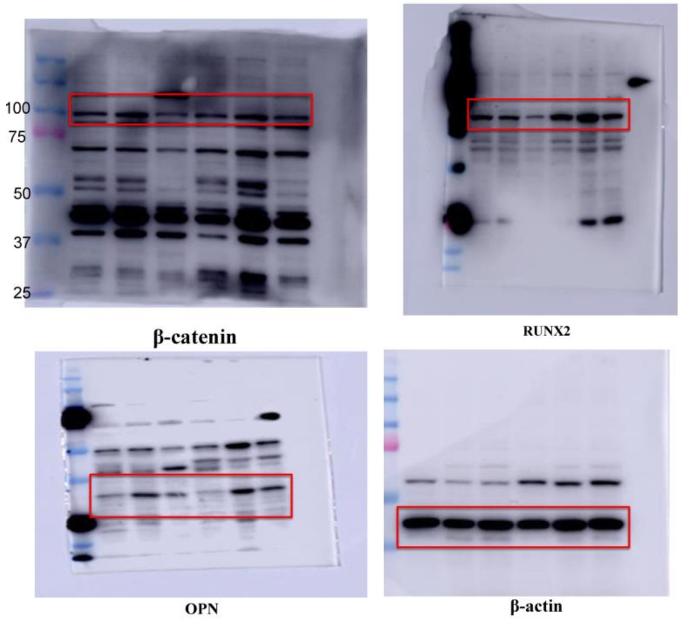

Figure S8 C

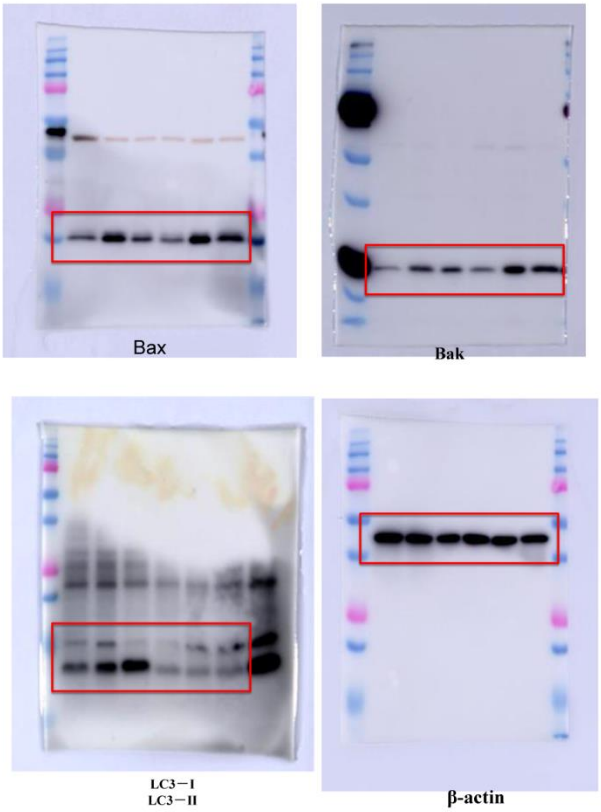

Supplement: Supplementary file 1 [file DataSheet_1.pdf]
